# Generative Adversarial Network and Chaotic Map-Based Multi-Layer Medical Image Encryption

**DOI:** 10.3390/s26144359

**Published:** 2026-07-09

**Authors:** Kaan Doğan Erdoğan, Nurettin Doğan

**Affiliations:** 1 Department of Property Protection and Security, Selcuk University, Konya 42400, Türkiye; 2 Faculty of Technology, Department of Computer Engineering, Selcuk University, Konya 42130, Türkiye; nurettin.dogan@selcuk.edu.tr

**Keywords:** medical image encryption, generative adversarial network, piecewise linear chaotic map, DNA complement transformation, bit-level zigzag permutation, noise vector key management, deep learning-based cryptography

## Abstract

One of the major challenges in securing medical image communication systems is the secure and efficient management of cryptographic key material. In this paper, we propose a multi-layer image encryption algorithm that addresses image security while reducing per-image key-storage and transmission overhead under a pre-shared protected-generator model. The proposed algorithm integrates a Generative Adversarial Network, a Piecewise Linear Chaotic Map, DNA complement operations, and bit-level zigzag permutation. A distinguishing feature of the proposed algorithm is that the key image is generated from an image-specific 100-dimensional noise vector, which serves exclusively as the input to the trained generator, while the chaotic parameters and diffusion materials are derived from the generated key image. In this approach, under the assumption of a pre-shared protected generator, transmitting only the image-specific 100-dimensional noise vector that bears no structural relationship to the key image reduces per-image key storage and transmission overhead. Comprehensive numerical evaluations were performed on eleven images, comprising both standard test images and medical images, to assess the security and robustness of the proposed algorithm. The experimental results demonstrate entropy values exceeding 7.996 bits, along with NPCR and UACI values of 99.60% and 33.46%, respectively. Adjacent pixel correlations are reduced to near-zero levels across all tested images. The proposed algorithm exhibits strong robustness against common attacks, including up to 75% cropping and 50% salt-and-pepper noise. The proposed algorithm achieves competitive performance compared with several existing encryption methods. Successful decryption requires the correct image-specific noise vector and the original trained generator.

## 1. Introduction

In recent years, diagnostic imaging has become an integral part of healthcare systems for determining appropriate treatments for patients. Medical images may contain sensitive personal information (e.g., medical record numbers and demographic data) [1,2] and are transmitted across networks such as telemedicine platforms and the Internet (e.g., cloud computing systems) [1,3]. When transmitted over unsecured networks, these sensitive healthcare records are at risk of unauthorized access and misuse [1,2,3]. Therefore, there is a need to develop reliable and computationally efficient encryption systems specifically designed to protect medical images.

Traditional encryption algorithms such as DES, IDEA, RSA, and other text-oriented cryptographic methods do not adequately address the specific characteristics of digital images, including large data volumes, high redundancy, and strong correlation among neighboring pixels [4,5,6]. Nonlinear approaches, particularly chaos-based image encryption schemes, have therefore attracted considerable attention in modern cryptography due to the pseudo-randomness, unpredictability, ergodicity, and high sensitivity of chaotic maps to initial conditions and control parameters [5,6]. Most chaos-based image encryption systems employ chaotic maps as pseudo-random sequence generators to perform two fundamental operations: pixel position permutation and pixel value diffusion [4,6,7]. Consequently, the dynamic properties of the selected chaotic map significantly affect the key space, statistical randomness, diffusion performance, and resistance of the encryption system to cryptanalytic attacks [5,6].

The need for increased security in cryptography has resulted in a growing diversity of chaotic algorithms. Creative efforts in biological computing have recently enabled the integration of cross-plane DNA operations with custom two-dimensional mapping by Zhang and Zhang, leading to the generation of exceptionally high levels of entropy [8]. Ding et al. further extended this direction by developing a four-dimensional multi-wing system to perform DNA-level shuffling via discrete wavelet decomposition based on the Sprott-A model [9]. Huang et al. employed a combination of hybrid Chebyshev–Tent/Sine–Tent–Cosine mapping with Fisher–Yates randomization to achieve robust statistical randomness in scrambling techniques [10], while Mathivanan and Maran designed a kolam-inspired shuffling technique based on a modified logistic cascade [11].

There has been a noticeable shift towards higher-dimensional algorithms due to the exploitation of spatial dependencies in the field of chaos-based cryptography. Examples of this trend are found in the work of Huang et al. [12] who used a drive-to-response mechanism to achieve three-dimensional asymptotic shape synchronization and the work of Zhang et al. [13] who created a variable structure hyperchaotic system using fractal principles with the VSDHS-CIEA protocol. Recently, efforts have also been directed toward connecting theoretical to practical implementations of cryptographic primitives and hardware such as the memristor-based multi-wing attractors developed by Wang et al. [14] and the delta-kicked oscillator integrated with PBKDF2-HMAC-SHA256 developed by Valencia et al. [15]. However, while chaos-based cryptosystems exhibit strong security profiles, a fundamental limitation exists on the operational capabilities of chaos-based cryptography that is dependent upon the mathematical unpredictability of the control parameters used to operate the system. This same sensitivity, while providing strong cryptographic performance, also creates substantial logistical burdens as it necessitates accurate preservation and secure transmission of the precise initial conditions used in order for correct decryption to occur. As such, this requirement increases the overhead associated with cryptographic key management.

Scientists have explored various methods to introduce stronger nonlinear behavior and diffusion characteristics into encryption algorithms through DNA computing. In many cases, binary pixel values are mapped into nucleotide sequences, followed by the application of biomolecular operations such as complementary base pairing to increase bit-level complexity [16,17]. Several studies incorporate DNA-based transformations into their encryption algorithms. For example, Ding et al. [9] employed wavelet decomposition together with DNA-level scrambling and bidirectional diffusion to construct a hybrid algorithm; Liu et al. [18] combined a hyperchaotic system with dynamic DNA coding and bit-level permutation to enhance both confusion and diffusion; and Li et al. [19] utilized a dual-chaotic structure with adaptive nucleotide encoding to establish a secure relationship between key generation and the original image, enabling block-level genetic transformations. Additionally, Dash et al. [20] proposed the Medisecure algorithm, which integrates GAN-based compression of medical data with DNA encoding for improved security. Al-Shargabi et al. [21] introduced the RDNA protocol, exploiting the stochastic properties of genetic bases to derive pseudorandom cryptographic keys from nucleotide structures. Wang et al. [22] developed a high-speed cryptosystem addressing the latency constraints of clinical imagery by employing a two-dimensional Chebyshev–Rulkov neuronal model together with rapid Hachimoji transcription and adaptive base-mutation mechanisms. Tang et al. [23] further improved the balance between compression and encryption by combining DNA encoding, S-box-based substitution, and block compressive sensing. Overall, these studies indicate that DNA-based transformations effectively function as nonlinear diffusion layers at the bit-level, significantly increasing the statistical complexity of encrypted outputs.

Significant advancements in image encryption have also been supported by deep learning techniques. Recent studies have incorporated neural networks into encryption frameworks for tasks such as compression, feature extraction, and cryptographic key generation. For example, Cheng et al. combined BP neural network-based compression with chaotic multi-image encryption, while Ding et al. proposed DeepKeyGen, a GAN-based stream cipher generator that generates private keys for medical image encryption and decryption [24,25]. Duggirala and Sathya created a hybrid architecture that utilized both CNNs, GANs, and autoencoders to provide secure medical image encryption in the Internet of Medical Things (IoMT) contexts [26]. Afzal et al. proposed a lightweight, IoT-oriented algorithm to provide selective security with an edge-based image classification system using hybrid chaotic encryption as part of this algorithm [27]. Wu et al. proposed an original algorithm for providing plausible deniability through the combination of large language models and reversible DNA transformations for content-aware image encryption [28]. Wang et al. created an original cryptosystem for securely transmitting facial images that are based on dlib facial feature recognition, combined with chaotic scrambling defined by an optimized memristor based neural network [29]. Gopalakrishnan et al. created the SCAM-MODEL; a combination of the Horsy Chess scrambling method and a deep learning-based cardiac MRI classification system [30]. In a related paper by Wang et al., a multi-image optical encryption algorithm using a physics-enhanced deep neural network was presented to address the challenges of conventional approaches [31]. Although deep learning-based encryption methods have shown promising results, their roles in existing frameworks remain largely task specific. Some approaches employ neural networks mainly for compression, feature extraction, or classification, whereas only a limited number of studies explicitly use deep learning models for cryptographic key generation. Moreover, when generated private keys or key images must be stored or transmitted together with encrypted medical data, additional key management and storage-security concerns may arise [24,25,27].

Hybrid and multi-stage image encryption schemes have been widely investigated to improve confusion, diffusion, and resistance to statistical or differential attacks by combining complementary cryptographic components within a single framework [32,33,34,35,36,37,38]. Some researchers are exploring multi-stage systems where several different cryptographic primitives could be used. For instance, Podder et al. proposed a cryptographic system that incorporates three types of cryptography: scrambling using the manipulation of array indexes; constructing a chaotic S-box; and creating a diffusion function using Hénon and Tent maps [32]. Kumar and Dua developed an exponential sine-cosine chaotic map to create batch encryption of a message by performing dynamic permutations, along with a DNA-assisted diffusion [33]. Ullah et al. developed an integrated system that combines an Arnold Cat Map rotation with a substitution S-box derived from a fractional-order hyperchaotic system and a permutation based on Hénon maps [34]. Safdar et al. developed an algebraic robust S-box for a cryptographic system as a nonlinear block cipher over a finite non-chain semi-local ring [35]. Inam et al. constructed a hybrid architecture by incorporating an error correction code and key sequences based on Hénon maps to enhance the reliability of transmitted data [36]. Wu et al. introduced a new protocol combining a three-dimensional hyperchaotic map with block compressive sensing and a randomization technique based on Peano curves that addresses the problem of inter-channel dependency [37]. Liu et al. developed a method of protecting multiple images at once by combining an extended logistic chaotic map with a directional shift transformation [38]. Together, these studies provide significant evidence that hybrid and multi-stage systems represent a very promising approach to producing high-quality, consistent, strong encryption performance by taking advantage of the complementary attributes of multiple types of cryptography.

Based on these studies, existing multi-stage image encryption frameworks can be broadly grouped into several categories: chaos-driven permutation–diffusion schemes, chaos–DNA hybrid schemes, S-box/substitution-enhanced architectures, compression-assisted encryption frameworks, and deep learning-supported encryption or key-generation methods. These studies demonstrate that combining multiple cryptographic primitives can improve confusion, diffusion, statistical randomness, and resistance to differential attacks. However, integrating GAN-based key synthesis, chaos-driven diffusion, DNA-level nonlinear transformation, and bit-level permutation within a single reversible architecture introduces additional technical challenges. The generated key image must be reproducible during decryption without explicit storage, the chaotic parameters must remain synchronized with the key-generation process, each transformation layer must preserve reversibility, and the contribution of each layer to confusion, diffusion, nonlinearity, and spatial decorrelation must remain technically separable. In this context, the proposed method differs from prior multi-stage approaches by using an image-specific 100-dimensional noise vector as the input to the protected GAN generator, while deriving the PWLCM parameters and diffusion materials from the generated key image, thereby linking the encryption layers through the GAN-generated key image under a compact key-management mechanism rather than relying on separately stored key images, independent chaotic seeds, or multiple unrelated secret parameters [24,25,32,33,34,35,36,37,38].

Additionally, the robustness of current chaos-based encryption systems has also been examined through cryptanalysis. Specifically, Amiri & Zaied created a method named DeepCryptanalysis, using a model based on a U-Net with deep learning that identifies and exploits weaknesses of chaos-based visual ciphers structurally in order to analyze them [39]. This finding underscores the importance of developing encryption algorithms with sufficiently complex key spaces and multi-layered security algorithms that can withstand both traditional and learning-based cryptanalytic threats.

Although numerous image encryption algorithms have been proposed, key generation and key management remain important limitations in many existing schemes. In chaos-based cryptosystems, encryption performance is strongly dependent on the precise initial conditions and control parameters, which must be preserved or shared accurately for successful decryption [5,6]. Similarly, deep learning-based key generation can produce highly random private keys, but storing or transmitting generated key images or model-derived private keys may introduce additional key management and storage-security concerns [25].

Although previous studies have investigated deep learning-based key generation, chaotic diffusion, DNA-based transformations, and bit-level permutation in different encryption contexts, their integration within a unified architecture combining a protected long-term generator with newly generated image-specific noise vectors remains insufficiently explored [16,17,25,32,33,34,35,36,37,38].

To address these limitations, we propose a multi-layer image encryption algorithm that leverages a Generative Adversarial Network (GAN), a Piecewise Linear Chaotic Map (PWLCM), DNA complement transformations, and bit-level zigzag permutation. The proposed algorithm is initiated by an image-specific 100-dimensional noise vector, which serves exclusively as the input to the trained GAN generator. The PWLCM parameters and diffusion materials are subsequently derived from the generated key image. Under the assumption that the trained generator is pre-shared and protected as a long-term secret component, only the low-dimensional image-specific noise vector needs to be retained for each encryption instance. By regenerating the same key image during decryption, this design eliminates the need to store or transmit the full-size generated key image, thereby reducing per-image key-storage and transmission overhead.

The proposed encryption process is implemented through an integrated sequence of operations. First, the original image is combined with the GAN-generated key image using a pixel-wise XOR operation, establishing initial confusion. The first 100 normalized values of the generated key image are then transformed into four real-valued control parameters using index-parity summation and positional segmentation, which serve as initial conditions for the PWLCM. The PWLCM generates a one-dimensional chaotic key stream with an initial value and a second chaotic RGB matrix. Subsequently, forward chained diffusion is applied using the generated chaotic materials. After diffusion, a DNA complement transformation is applied at the 2-bit block level to provide a reversible bit-level modification. Subsequently, a channel-specific zigzag permutation is performed at the bit-plane level to disrupt spatial and inter-channel correlations. Finally, bidirectional chained diffusion is applied along the image rows and columns.

The main contributions of this paper are as follows:

(1) Image-Specific Noise-Vector-Based Key Management: This study proposes a unified key management mechanism based on an image-specific noise vector; however, the vector itself carries no meaningful structural information about the generated key image. Under a pre-shared protected-generator assumption, a newly generated noise vector is used for each encryption instance, thereby reducing per-image key storage and transmission overhead.
(2) GAN-Based Dynamic Key Generation: This study proposes a dynamic key generation mechanism based on a trained DCGAN that generates 256 × 256 × 3 synthetic medical images as cryptographic keys from image-specific random noise vectors. During the decryption phase, the same noise vector is used to reconstruct the identical key image, eliminating the need to store the full-size key image.
(3) Multi-Stage Chaotic Diffusion Using Key-Image-Derived Control Parameters: This study employs a forward chained diffusion process followed by bidirectional row and column diffusion. Four control parameters are derived from the generated key image using index-parity summation and positional segmentation. These parameters serve as initial seeds for the PWLCM and generate a chaotic key stream with an initial value and a second chaotic RGB matrix.
(4) Reversible Bit-Level Modification Through DNA Complement Transformation: This study introduces a reversible bit-level transformation using a DNA complement operation applied to each 2-bit block, based on biological complementarity rules (A↔T; C↔G).
(5) Channel-Specific Bit-Level Zigzag Permutation: This study performs a channel-specific bit-level zigzag permutation to reduce inter-channel dependencies and minimize spatial correlation. The RGB channels are first converted into bit matrices and then permuted using different traversal patterns (serpentine, spiral, and mirrored diagonal).
(6) Multi-Layer Reversible Algorithm: This study proposes a multi-layer image encryption model that integrates generative confusion, multi-stage chaotic diffusion, DNA sequence operations, and bit-level permutation. The resulting algorithm requires the exact image-specific noise vector and the original trained generator for this purpose, while possession of either component alone is insufficient for successful decryption.

This paper is organized as follows. Section 2 provides a detailed description of the proposed cryptosystem, including the GAN, the PWLCM-based diffusion layer, DNA complement operations, and the zigzag permutation strategy. Section 3 presents experimental results and security analysis, including histogram analysis, quality metrics (MSE, PSNR, and SSIM), information entropy, differential analysis (NPCR and UACI), correlation analysis, chosen-plaintext attack evaluation, and robustness against cropping and noise attacks. Section 4 concludes the paper and discusses the implications of the proposed approach.

## 2. Proposed Method

**Figure 1:**
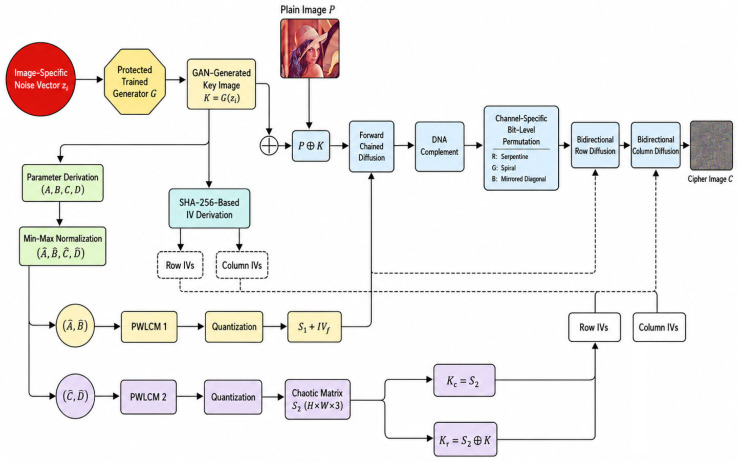
Block diagram of the proposed multi-layer image encryption algorithm.

As illustrated in Figure 1, the proposed multi-layer image encryption algorithm integrates Generative Adversarial Network (GAN)-based key synthesis, Piecewise Linear Chaotic Map (PWLCM)-driven diffusion, DNA complement transformation, and channel-specific bit-level permutation. At the core of the proposed algorithm lies a 100-dimensional noise vector that is used exclusively as the input of the trained GAN generator. For each image-encryption instance, a newly generated noise vector is used to produce an image-specific key image. The remaining chaotic parameters and diffusion materials are subsequently derived from the generated key image rather than directly from the noise vector.

The process used to generate the key image is as follows: A trained GAN generator network produces a 256 × 256 × 3 synthetic RGB key image from a randomly initialized noise vector. The generator network is structured with one dense (fully connected) layer and six transposed convolutional layers. The generated key image is not stored; instead, only the corresponding noise vector is retained separately from the trained generator model. During decryption, the same noise vector is input to the same trained generator to reproduce the identical key image. Since a different noise vector is assigned to each image-encryption instance, the resulting key image and the associated diffusion materials also vary from one image to another.

Following key generation, the protocol introduces an initial confusion stage by applying a pixel-wise XOR operation between the plain image and the GAN-generated key image. Four real-valued control parameters are then calculated from the normalized intensity values of the generated key image using index-parity summation and positional segmentation. These parameters are normalized to the valid PWLCM interval and used to generate the chaotic sequences required by the diffusion stages. The first PWLCM sequence forms an image-length key stream and an initial value for a forward chained diffusion process. In this process, each encrypted byte depends on the corresponding input byte, the chaotic key-stream value, and the previously produced cipher byte, thereby propagating local plaintext changes through the subsequent data positions.

After the forward diffusion stage, a DNA complement operation is applied channel-specifically at the 2-bit block level. Based on biological complementarity rules (A↔T, C↔G), the lower-order bit of each 2-bit block is inverted. This operation provides a reversible bit-level modification before the permutation and spatial diffusion stages. Subsequently, a channel-specific bit-level permutation is applied after each RGB channel has been expanded into a bit matrix. The red, green, and blue channels are permuted using serpentine, spiral, and mirrored diagonal traversal patterns, respectively. In addition, circular shifts are applied to the higher-order bit rows of the red channel. These operations redistribute the bit positions across the image and reduce the local spatial dependencies among neighboring pixels.

A second PWLCM sequence is reshaped into an RGB key matrix and used in the remaining diffusion stages. The row-diffusion key is obtained by combining this chaotic matrix with the GAN-generated key image, whereas the column-diffusion key is formed directly from the chaotic matrix. Deterministic initial values for the row and column operations are also derived from the generated key image. Bidirectional chained diffusion is then applied first along the image rows and subsequently along the image columns. During each forward and backward pass, the current output depends on the corresponding input value, the associated diffusion-key value, and the previously generated value in the direction of processing. This structure increases the propagation of small plaintext changes across both spatial dimensions.

To recover the original data, the decryption process reverses the sequence of operations used during encryption. First, the stored noise vector is fed into the same trained GAN generator to reproduce the identical key image. The PWLCM control parameters, chaotic sequences, initial values, and row- and column-diffusion materials are then recalculated from the regenerated key image. The column diffusion and row diffusion stages are inverted in reverse order, followed by the inverse bit-level permutation. The DNA complement operation is subsequently reapplied due to its self-inverse property. The forward chained diffusion is then reversed using the regenerated chaotic key stream and initial value. Finally, the original image is reconstructed by applying a pixel-wise XOR operation with the regenerated GAN key image. Consequently, successful decryption requires both the exact image-specific noise vector and the original trained generator model.

### 2.1. Generative Adversarial Network

A Generative Adversarial Network (GAN), specifically a Deep Convolutional Generative Adversarial Network (DCGAN), is used to generate key images using a generator and a discriminator within an adversarial framework. The generator takes a random noise vector sampled from a standard Gaussian distribution (100-dimensional) and produces a synthetic RGB image of size 256 × 256 × 3. The generator is structured with a fully connected layer followed by multiple transposed convolutional layers. Batch normalization and Rectified Linear Unit (ReLU) activations are used to stabilize the training of these layers. Finally, the output of the generator is scaled using a Tanh activation function, resulting in pixel values within the range [−1, 1].

**Figure 2:**
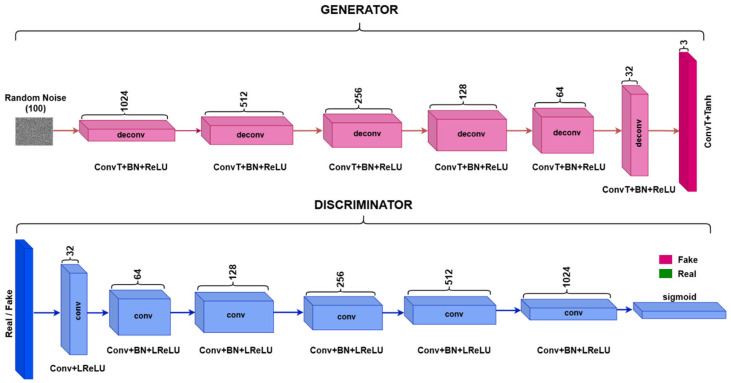
Architecture of the DCGAN model used for key image generation.

The discriminator evaluates the authenticity of generated and real images through progressive downsampling to produce a classification output. The overall architecture (including both components) is illustrated schematically in Figure 2.

For training, a publicly available dataset of Acute Lymphoblastic Leukemia (ALL) images was used. The dataset contains 3256 peripheral blood smear (PBS) microscopy images from 89 patients [30]. It includes both benign haematogones and malignant ALL subtypes (Early Pre-B, Pre-B, and Pro-B). To preserve morphological characteristics while ensuring consistent input dimensions, all images are resized to 256 × 256.

The DCGAN was trained using the Adam optimizer with a learning rate of 1 × 10^−4^, β_1_ = 0.5, and β_2_ = 0.999 for both the generator and discriminator networks, consistent with standard GAN training practice [29,40]. A batch size of 16 was employed throughout training, and the model was trained for 20 epochs. The generator checkpoint saved at the final epoch was retained for subsequent key image generation. The final generator checkpoint was selected because the objective of the proposed cryptosystem is not photorealistic medical image synthesis, but the generation of diverse and structurally irregular key images suitable for encryption. Therefore, model selection was based on output diversity, deterministic reproducibility from the same noise vector, and cryptographic key sensitivity rather than visual realism or adversarial convergence alone. All experiments were performed on a Windows 11 Pro with an AMD Ryzen 7 5825U CPU (8 cores, 16 threads), AMD Radeon (TM) Graphics, and 16 GB of system memory. The implementation was developed in Python using PyTorch (Python 3.13.9, PyTorch 2.7.0). Since CUDA acceleration was unavailable in the current runtime environment, all reported runtime measurements were obtained on the CPU.

The number of training epochs is intentionally limited to 20 to reduce the risk of overfitting and memorization, thereby ensuring that the generated key images remain sufficiently unpredictable for cryptographic use. Unlike conventional GAN training objectives that prioritize stable adversarial convergence, the proposed system deliberately operates in a partially trained regime, in which the generator produces diverse and structurally irregular synthetic images. This property is cryptographically desirable, as it ensures that the generated key images do not exhibit predictable structural patterns that could be exploited in statistical or chosen-plaintext attacks. Accordingly, full adversarial convergence was not used as the primary stopping criterion. Instead, training stability was assessed from the cryptographic perspective by examining whether the generator produced reproducible outputs for identical noise vectors and sufficiently different key images for small perturbations in the noise vector. This behavior was further evaluated through the key sensitivity and differential-security analyses reported in Section 3.7.

In the context of the proposed cryptosystem, limited output diversity would manifest as the generation of similar key images from distinct noise vectors, thereby reducing the effective diversity of the image-specific key material. The key-sensitivity analysis presented in Section 3.7 evaluates this issue from a cryptographic perspective. The reported results show that the tested perturbations in the noise vector produce substantially different cipher images, with NPCR values exceeding 99.4% across the evaluated channels. These findings provide empirical evidence that the generator outputs used in the experiments exhibit sufficient sensitivity to changes in the noise vector. However, this analysis is interpreted as a cryptographic key-sensitivity evaluation rather than as a formal assessment of mode collapse in the generative model.

The generator network G is optimized through the following adversarial loss function, as defined in Equation (1):

(1) $$L_{G}=-E_{\left\{z∼p_{\left\{noise\right\}\left(z\right)}\right\}}\left[\log\left(D\left(G\left(z\right)\right)\right)\right]$$

where z denotes the noise vector and D represents the discriminator network. In practice, this objective is implemented using Binary Cross Entropy (BCE) loss, as expressed in Equation (2):

(2) $$L_{G}=BCE\left(D\left(G\left(z\right)\right),1\right)$$

The discriminator loss function is formulated as given in Equation (3):

(3) $$L_{D}=E_{\left\{y∼p_{data}\left(y\right)\right\}}\left[\log D\left(y\right)\right]+E_{\left\{z∼p_{noise}\left(z\right)\right\}}\left[\log\left(1-D\left(G\left(z\right)\right)\right)\right]$$

which is implemented as shown in Equation (4):

(4) $$L_{D}=BCE\left(D\left(y\right),1\right)+BCE\left(D\left(G\left(z\right)\right),0\right)$$

After completing adversarial training, the generator is used to produce synthetic RGB images from 100-dimensional noise vectors. In the proposed cryptosystem, these generated outputs are employed exclusively as image-specific cryptographic key images rather than for diagnostic interpretation or patient representation. By using the generator, a newly generated image-specific 100-dimensional noise vector is fed into the generator, and it deterministically generates a synthetic image, which serves as the image-specific key image and the source of the downstream chaotic diffusion material, rather than storing the actual full-size key image. The generated key image is not stored. Instead, the image-specific noise vector is retained separately from the trained generator, which is maintained as a protected long-term system component. In order to decrypt the encrypted data, the receiver provides the same noise vector used during encryption to the protected generator. This reproduces the identical key image required for decryption. Knowledge of the noise vector alone does not reveal the internal parameters of the trained generator. Even if the generator architecture, training dataset, and training configuration are known, an independently trained generator will generally obtain a different set of learned parameters and therefore produce a different output for the same noise vector. Since successful decryption requires the byte-identical key image generated by the original model checkpoint, both the exact image-specific noise vector and the original trained generator are required.

It is important to note that the cryptographic performance of the proposed system does not rely on semantic consistency between the GAN training domain and the target image domain. In the proposed architecture, the generator serves exclusively as a deterministic key-image generation function, G: ℝ^100^ → ℝ^256×256×3^. The characteristics of the training dataset influence the visual appearance of the generated key image K = G(z), but the generator does not receive, analyze, or adapt to the plaintext image during encryption. Therefore, the target image domain, whether a standard benchmark image or a medical image, has no direct effect on the generated key material.

The security of the proposed system depends on the exact reproducibility of K from the same latent vector z, the sensitivity of the generated key material to small perturbations in z, and the subsequent cryptographic transformations applied by the multi-layer encryption pipeline. In this respect, the GAN component functions as a compact and reproducible key-generation mechanism rather than as a domain-specific image encryptor. The use of standard benchmark images such as Lena, Mandrill, Peppers, and Sailboat in the experimental evaluation follows established practice in image encryption research and enables direct comparison with existing methods. Medical images are also included to confirm that the proposed method maintains consistent encryption behavior across different image types.

A critical design consideration of the proposed system concerns the confidentiality requirements of the trained generator G. In the proposed architecture, G is treated as a protected long-term secret component of the cryptographic system and is not intended for public distribution. The image-specific noise vector z is maintained separately from the trained generator and is renewed for each encryption instance. This design decision is consistent with the principle of defense in depth. Successful decryption requires the simultaneous possession of two separately protected secret components: the exact image-specific noise vector z and the original trained generator G. The absence of either component prevents the regeneration of the key image K = G(z) and the associated chaotic diffusion material.

It is important to distinguish the security model of the proposed system from traditional key storage approaches. In conventional symmetric encryption, the full-size key image must be stored or transmitted, introducing the risk of database-level compromise. In the proposed system, only the compact 100-dimensional noise vector z is transmitted, and the key image K = G(z) is never stored or transmitted in any form. The trained generator G is pre-shared between communicating parties through a secure channel prior to communication, analogous to pre-shared secret material in symmetric cryptographic systems. This one-time secure distribution of G reduces the need for repeated transmission of full-size key images, since G does not vary between encryption sessions and can be protected using established secure communication mechanisms such as TLS or public-key infrastructure.

While the confidentiality of G introduces an additional system-management requirement compared with purely noise-vector-based schemes, this complexity is offset by the reduction in per-image key-storage and transmission overhead. The architecture, training dataset, and training procedure of the generator may be known without revealing the exact learned parameter set of the original model. Owing to the stochastic initialization and optimization processes involved in GAN training, independently retraining the same architecture on the same dataset does not generally reproduce the same parameter values or the same deterministic mapping from z to K. Therefore, reproducing the byte-identical key image required for decryption depends on possession of the original trained generator checkpoint rather than on knowledge of its general architecture or training data alone.

### 2.2. Piecewise Linear Chaotic Map (PWLCM)

The PWLCM is an effective and straightforward mathematical method of creating a highly unpredictable random sequence of numbers. The PWLCM is recognized by strong chaotic behavior (as indicated by a positive Lyapunov exponent) and is also distinguished for its high information entropy; thus, it has been used extensively as a source of randomness for the generation of random image encryption/decryption keys [5]. The PWLCM provides a much larger region of chaos and much greater complexity than the standard logistic map when iterated over a unit interval. As a result, these characteristics make the PWLCM a suitable basis for designing secure image encryption systems. The PWLCM is described in Equation (5) [5,6]:

(5) $$f_{p}\left(u\right)=\left\{\begin{array}{cc} \frac{u}{p}, & \mathrm{if}\;0\leq u<p,\\ \frac{1-u}{1-p}, & \mathrm{if}\;p\leq u\leq 1, \end{array}\right.$$

**Figure 3:**
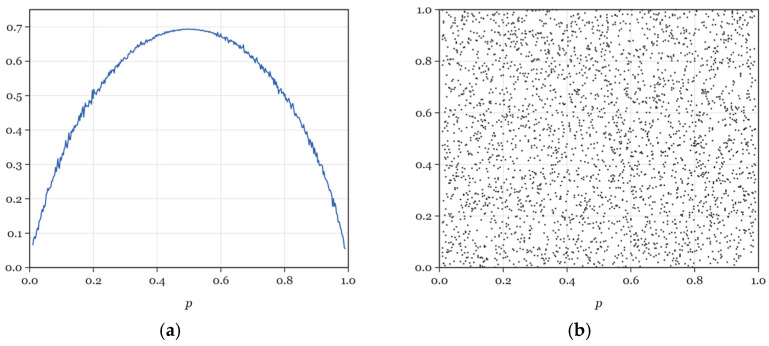
Chaotic behavior analysis of PWLCM across the control: (**a**) Lyapunov exponent and (**b**) bifurcation diagram.

The PWLCM used in this algorithm can be regarded as a generalization of the standard tent map, which is a special case occurring at *p* = 0.5. The PWLCM is initialized using parameters derived from the GAN-generated key image, which are subsequently normalized to the interval [0.1, 0.9] via min-max normalization as described in Equation (7), ensuring strict compliance with the valid chaotic domain. The first 100 iterations are discarded to eliminate transient effects before generating the main chaotic sequence. The bifurcation diagram and Lyapunov exponent (LE) analysis of the PWLCM are presented in Figure 3, showing that the Lyapunov exponent remains positive over the parameter range used in this study, indicating consistent chaotic behavior across the domain.

For each instance of configuration of the PWLCM, the parameters that are required to initialize and control it are not predetermined. Rather, they are calculated from the first 100 normalized intensity values of the GAN-generated key image. After the GAN-generated key image is flattened in interleaved RGB order, its first 100 intensity values are normalized by division by 255 and defined as $x=\{x_{1},x_{2},\ldots ,x_{100}\}$. Four parameters are computed using summation-based expressions, as defined in Equation (6):

(6) $$A=\frac{\sum_{k=1}^{50}x_{2k}}{10},B=\frac{\sum_{k=1}^{50}x_{2k-1}}{10},C=\frac{\sum_{i=1}^{50}x_{i}}{10},D=\frac{\sum_{i=51}^{100}x_{i}}{10}$$

By this formulation, four intermediate parameters are derived from different subsets of the GAN-generated key image.

**Figure 4:**
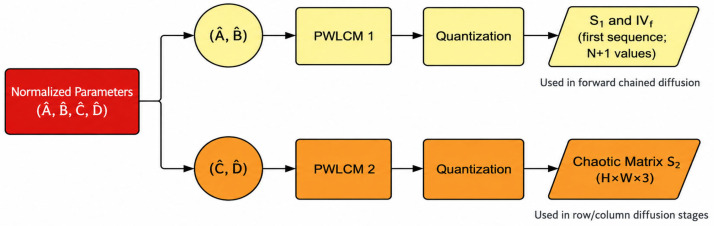
Generation of PWLCM sequences using parameter pairs derived from the GAN-generated key image.

Since the raw parameters computed via Equation (6) may exceed the unit interval as a result of the summation operations applied to the normalized key-image values, a min-max normalization step is applied to map each parameter into the interval [0.1, 0.9], as defined in Equation (7):

(7) $${\hat{\theta }}_{i}=0.1+\frac{\theta_{i}-\theta_{min}}{\theta_{max}-\theta_{min}}\times 0.8$$

where θ∈A,B,C,D, and $\theta_{min}$, $\theta_{max}$ denote the minimum and maximum values among the four raw parameters. In the degenerate case where all parameters are equal, each is assigned the midpoint value of 0.5. This transformation guarantees that all PWLCM initial conditions and control parameters remain strictly within the valid chaotic domain (0, 1). The normalized parameters serve as the initial conditions and control parameters of the PWLCM for the two chaotic sequences illustrated in Figure 4. The normalized parameter pair ($\hat{A},\hat{B}$), where A is used as the initial condition and B as the control parameter, produces the first chaotic sequence, which provides the key stream and initial value required by the forward chained diffusion stage. Similarly, the normalized parameter pair ($\hat{C},\hat{D}$), where C is used as the initial condition and D as the control parameter, produces the second chaotic sequence, which provides the key material required by the row and column diffusion stages. For an RGB image of size H×W, the total number of byte values is N=H×W×3. Accordingly, the first chaotic sequence contains N + 1 values, whereas the second chaotic sequence contains N values. The continuous outputs are converted to discrete values by taking the floating-point number and multiplying it by 256, followed by the use of the floor function to generate standard 8-bit integer values within the defined range. The first quantized sequence is retained in one-dimensional interleaved RGB form for the forward chained diffusion stage, whereas the second sequence is reshaped into an H×W×3 chaotic RGB matrix for the subsequent row and column diffusion stages.

After the first 100 iterations are discarded, the first PWLCM produces a chaotic sequence denoted by $Q^{\left(1\right)}\;=\;\{{q_{1}}^{\left(1\right)},\;{q_{2}}^{\left(1\right)},\;\ldots ,\;q_{N+1}^{\left(1\right)}\}.$ The first N values are quantized to obtain the key stream $S_{1}$, whereas the final value is used as the initial value $IV_{f}\;$of the forward chained diffusion stage, as defined in Equation (8):

(8) $$S_{\left(1,n\right)}=\lfloor 256q_{n}^{\left(1\right)}\rfloor ,\;n=1,2,\ldots ,N,\;IV_{f}=\lfloor 256q_{N+1}^{\left(1\right)}\rfloor .$$

Similarly, the second PWLCM produces a sequence $Q^{\left(2\right)}=\left\{q_{1}^{\left(2\right)},q_{2}^{\left(2\right)},\ldots ,q_{N}^{\left(2\right)}\right\}$. Its values are quantized according to Equation (9):

(9) $$S_{2,n}=\left\lfloor 256q_{n}^{\left(2\right)}\right\rfloor ,\;n=1,2,\ldots ,N.$$

The resulting sequence is reshaped in interleaved RGB order to form the chaotic matrix $S_{2}$, as expressed in Equation (10):

(10) $$S_{2}={reshape}_{H\times W\times 3}\left({\left\{S_{2,n}\right\}}_{n=1}^{N}\right).$$

The key stream $S_{1}$ and the initial value $IV_{f}\;$are used in the forward chained diffusion stage, whereas the matrix $S_{2}$ is used to construct the key materials required by the subsequent bidirectional row and column diffusion stages.

### 2.3. Chained Diffusion

The proposed algorithm employs a dual diffusion structure consisting of two complementary stages. The first stage applies forward chained diffusion to the interleaved RGB byte sequence after the initial XOR operation with the GAN-generated key image. The second stage applies bidirectional chained diffusion along the image rows and columns after the DNA complement and bit-level permutation operations. In both stages, each output value depends on the corresponding input value, the associated chaotic key value, and a previously generated output value in the direction of processing.

In the first diffusion stage, the result of the pixel-wise XOR operation between the plain image and the GAN-generated key image is flattened in interleaved RGB order. The resulting byte sequence is represented as $M=\left\{M_{1},M_{2},\ldots ,M_{N}\right\}$. Using the key stream $S_{1}$ and the initial value $IV_{f}\;$generated in Section 2.2, the forward-diffused sequence $F=\left\{F_{1},F_{2},\ldots ,F_{N}\right\}$ is calculated as defined in Equation (11):

(11) $$F_{1}=\left(M_{1}+S_{1,1}+IV_{f}\right)\;\mathrm{mod}256,\;F_{n}=\left(M_{n}+S_{1,n}+F_{n-1}\right)\;\mathrm{mod}256,\;\;n=2,3,\ldots ,N.$$

Here, $M_{n}$ denotes the n-th byte of the interleaved RGB sequence, $S_{1,n}$ represents the corresponding value of the first PWLCM-derived key stream, and $F_{n-1}$ is the previously generated diffusion output. Therefore, except for the first value initialized by $IV_{f}$, each output byte depends on both the current input and the preceding output value.

During decryption, the original XOR-masked sequence is recovered by reversing the modular additions, as expressed in Equation (12):

(12) $$M_{1}=\left(F_{1}-S_{1,1}-IV_{f}\right)\;\mathrm{mod}256,\;M_{n}=\left(F_{n}-S_{1,n}-F_{n-1}\right)\;\mathrm{mod}256\;,\;\;n=2,3,\ldots ,N.$$

In the second diffusion stage, the chaotic RGB matrix $S_{2}$ is used to generate the row- and column diffusion keys. The row key is obtained by combining $S_{2}$ with the GAN-generated key image, while the column key is defined directly by $S_{2}$, as given in Equation (13):

(13) $$K_{r}=S_{2}⊕K,\;\;K_{c}=S_{2.}$$

Here, $K_{r}$ and $K_{c}$ are H×W×3 matrices used in the bidirectional row and column diffusion stages, respectively.

To obtain the initial values used in the bidirectional diffusion passes, the byte representation of the GAN-generated key image K is concatenated with fixed row and column labels and processed using SHA-256. The first 64 bits of the resulting hashes are used as the base values $B_{r}$ and $B_{c}$. The forward and backward initial values are then calculated as follows:

(14) $$IV_{y,c}^{r,f}=\left(B_{r}+17y+53c\right)\;\mathrm{mod}256,\;IV_{y,c}^{r,b}=\left(B_{r}+97y+29c\right)\;\mathrm{mod}256,\;IV_{x,c}^{c,f}=\left(B_{c}+31x+41c\right)\;\mathrm{mod}256,\;IV_{x,c}^{c,b}=\left(B_{c}+73x+19c\right)\;\mathrm{mod}256.$$

Here, y, x, and c denote the row, column, and color-channel indices, respectively. These values provide separate initialization for each row, column, channel, and processing direction.

The second diffusion stage begins with bidirectional processing along the image rows. The input obtained after the DNA complement and bit-level permutation operations is denoted by Z. For each row y and color channel c, the forward row pass is calculated as

$$R_{y,0,c}^{f}=\left(Z_{y,0,c}+K_{r,y,0,c}+IV_{y,c}^{r,f}\right)\;\mathrm{mod}256,\;R_{y,x,c}^{f}=\left(Z_{y,x,c}+K_{r,y,x,c}+R_{y,x-1,c}^{f}\right)\;\mathrm{mod}256\;,\;\;x=1,2,\ldots ,W-1.$$

The resulting values are then processed in the reverse direction as follows:

(15) $$R_{y,W-1,c}^{b}=\left(R_{y,W-1,c}^{f}+K_{r,y,W-1,c}+IV_{y,c}^{r,b}\right)\;\mathrm{mod}256,\;R_{y,x,c}^{b}=\left(R_{y,x,c}^{f}+K_{r,y,x,c}+R_{y,x+1,c}^{b}\right)\;\mathrm{mod}256\;,\;\;x=W-2,W-3,\ldots ,0.$$

Here, $R^{f}$ and $R^{b}\;$denote the forward- and backward-row diffusion outputs, respectively. The resulting matrix $R^{b}\;$is subsequently used as the input of the column-diffusion stage.

The row-diffused matrix $R^{b}\;$is then processed bidirectionally along the image columns. For each column x and color channel c, the forward column pass is calculated as

$$C_{0,x,c}^{f}=\left(R_{0,x,c}^{b}+K_{c,0,x,c}+IV_{x,c}^{c,f}\right)\;\mathrm{mod}256,\;C_{y,x,c}^{f}=\left(R_{y,x,c}^{b}+K_{c,y,x,c}+C_{y-1,x,c}^{f}\right)\;\mathrm{mod}256\;,\;\;y=1,2,\ldots ,H-1.$$

The backward column pass is subsequently applied as follows:

(16) $$C_{H-1,x,c}^{b}=\left(C_{H-1,x,c}^{f}+K_{c,H-1,x,c}+IV_{x,c}^{c,b}\right)\;\mathrm{mod}256,\;C_{y,x,c}^{b}=\left(C_{y,x,c}^{f}+K_{c,y,x,c}+C_{y+1,x,c}^{b}\right)\;\mathrm{mod}256\;,\;\;y=H-2,H-3,\ldots ,0.$$

Here, $C^{f}$ and $C^{b}$ denote the forward- and backward-column diffusion outputs, respectively. The final matrix $C^{b}$ constitutes the cipher image produced by the dual diffusion structure.

During decryption, the column-diffusion stage is reversed first, followed by the row-diffusion stage. The modular additions are inverted by modular subtraction using the same key matrices and initial values, and the operations are performed in the reverse order of encryption. The recovered matrix is therefore identical to the input of the second diffusion stage when the correct key material is regenerated.

### 2.4. DNA Encoding

A DNA strand consists of four nucleobases at the molecular level: A (adenine), T (thymine), C (cytosine), and G (guanine), which follow the Watson–Crick base-pairing rules: A pairs with T and C pairs with G. Modern image encryption systems increasingly leverage these properties by using operations derived from DNA sequences within encryption algorithms [17]. In these algorithms, image data is mapped to a DNA-based representation by assigning two digital bits to each nucleotide [16].

DNA has four types of bases that can be encoded in various ways. Only encodings that adhere to the rules of complementary pairing are considered for DNA-based image representations. Table 1 presents the encoding rules used in this paper, where binary values (00, 01, 10, and 11) are mapped to the corresponding nucleotides A, T, C, and G. To introduce an additional reversible bit-level transformation, operations derived from DNA complement rules are applied. Unlike traditional pixel-level approaches, these operations directly affect the binary representation of each pixel. Consequently, after the initial XOR operation and forward chained diffusion, this DNA-level transformation further modifies the binary representation of the pixel values before the subsequent bit-level permutation stage.

By partitioning the 8-bit pixel intensity values into four distinct 2-bit segments, the system maps these blocks to the corresponding DNA nucleotides (A, T, C, and G). Each encoded base undergoes a complement transformation governed by Watson–Crick complementarity rules. A sample RGB pixel is illustrated in Table 2. This bit-level transformation enhances the randomness of the pixel values and produces a more randomized bit distribution for the subsequent permutation stage. The original data can be accurately restored during decryption due to the self-inverse property of the DNA complement operation.

Let the position of a pixel be denoted by its row and column indices. Here, i denotes the row index of the pixel, j denotes the column index, and k represents the color channel index. Thus, the pixel value corresponding to the k -th channel is denoted by as P(i,j,k). This value is an integer in the range [0, 255] and is represented as an 8-bit binary sequence, as defined in Equation (17):

(17) $$B\left(i,j,k\right)={\left(b_{7}b_{6}b_{5}b_{4}b_{3}b_{2}b_{1}b_{0}\right)}_{2}$$

where $b_{m}\in \{0,1\}$ represents the m-th bit in the binary representation.

To perform the DNA-based operation, the binary sequence is divided into four 2-bit sub-blocks, as expressed in Equation (18):

(18) $$B\left(i,j,k\right)\to T\left(i,j,k\right)=\left\{t_{3},t_{2},t_{1},t_{0}\right\}\;t_{m}=\left(b_{2m+1},b_{2m}\right),t_{m}\in \left\{00,01,10,11\right\},m=0,1,2,3$$

Each 2-bit sub-block is symbolically mapped to a DNA base according to the fixed encoding rule adopted in this study, as given in Equation (19):

(19) 00↔A,01↔T,10↔C,11↔G

Based on the biological complementarity principle between DNA bases (A↔T, C↔G), a DNA complement transformation is defined for each 2-bit block, as formulated in Equation (20):

(20) $$f\left(t_{m}\right)=t_{m}⊕01$$

In this expression, ⊕ denotes the bitwise XOR operation. Unlike the data-dependent XOR operations applied in the chaotic diffusion stages, this transformation applies a constant mask, deterministically inverting the lower-order bit within each 2-bit block. Specifically, a fixed binary mask 01 is applied via a bitwise XOR operation to each 2-bit block, implementing the Watson–Crick complementarity rule at the bit-level.

After the complement operation, the four transformed 2-bit blocks are concatenated to reconstruct the 8-bit binary sequence. To conclude the transformation, the algorithm directly maps the reconstructed binary string to its corresponding decimal pixel intensity using Equation (21):

(21) $$P’\left(i,j,k\right)=\sum_{m=0}^{3}f\left(t_{m}\right)\cdot 2^{2m}$$

where $P^{’}(i,j,k)$ indicates the transformed pixel intensity value after DNA-based complement processing.

Since the transformation satisfies the self-inverse property given in Equation (22):

(22) $$f\left(f\left(t_{m}\right)\right)=t_{m}$$

as the operation is self-inverse, repeating the same transformation during decryption enables recovery of the original pixel value.

### 2.5. Channel Specific Bit Level Zigzag Scan

Image encryption algorithms often use scanning techniques, such as zigzag scanning, to reduce the high correlation between neighboring pixels. However, as this spatial permutation is limited to the pixel-level, the underlying bit-plane structure remains unchanged and may inadvertently leak statistical characteristics, exposing the cryptosystem to chosen-plaintext vulnerabilities [41].

In this research, a bit-level permutation technique is used to mitigate these vulnerabilities. Specifically, instead of the conventional (H × W) RGB image representation, the image is converted into a (H × 8) × W bit matrix, and bit-level permutation is performed within this matrix, disrupting both spatial positions and bit values of the original data to establish a dual-layer permutation mechanism [42].

**Figure 5:**
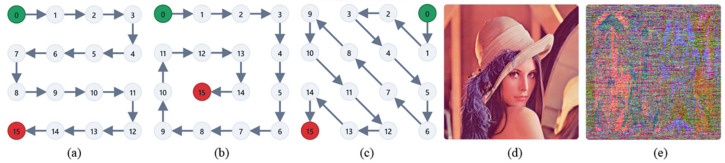
Channel-specific bit-level zigzag scan method: (**a**) serpentine traversal for the red channel, (**b**) clockwise spiral traversal for the green channel, (**c**) mirrored diagonal top-right-to-bottom-left traversal for the blue channel, (**d**) sample plain image, and (**e**) image after bit-level permutation.

In addition, a channel-specific permutation is implemented for each of the three channels of the RGB image, as illustrated in Figure 5. The scrambling process permutes the red, green, and blue bit matrices using different traversal patterns (the red bit matrix is permuted using a serpentine traversal pattern, the green bit matrix is permuted using a clockwise spiral traversal pattern, and the blue bit matrix is permuted using a mirrored diagonal top-right-to-bottom-left traversal pattern). The serpentine pattern alternates the horizontal scanning direction between successive rows, the spiral pattern follows a boundary-to-center traversal, and the mirrored diagonal pattern scans the bit matrix along diagonals from the top-right toward the bottom-left. After the serpentine permutation of the red-channel bit matrix, an additional circular shift is applied to its four higher-order bit rows. For a bit-matrix row indexed by r, the shift amount is calculated as $\left(3r\right)\mathrm{mod}W$, and the row is circularly shifted to the right by this value. The same shift is reversed before applying the inverse permutation during decryption. Ultimately, each channel is processed differently during the scrambling process, which effectively reduces inter-channel correlations.

This channel-specific differentiation reduces structural similarity across RGB components and enhances permutation effectiveness in the final encrypted image while preserving full reversibility.

Let the intermediate encrypted image be

(23) $$I\in {\left\{0,\ldots ,255\right\}}^{H\times W\times 3}$$

and let $I^{\left(I\right)}\in \{0,\ldots ,255{\}}^{H\times W}$, I∈{R,G,B}, denote its color channels. Each channel is first transformed into an expanded binary matrix, as defined in Equation (24):

(24) $$B^{\left(c\right)}=B\left(C^{\left(c\right)}\right),B^{\left(c\right)}\in {\left\{0,1\right\}}^{8H\times W}$$

where every pixel is decomposed into its 8-bit representation in MSB-to-LSB ordering. For each channel, a traversal-dependent permutation function $\pi_{m_{c}}$ is generated according to the selected zigzag type $m_{c}$. The permutation is then applied to the vectorized bit matrix, as expressed in Equation (25):

(25) $${\tilde{B}}^{\left(c\right)}\left(i\right)=B^{\left(c\right)}\left(\pi_{m_{c}}\left(i\right)\right),i=0,\ldots ,8HW-1$$

where the bit matrix is treated in row-major order. The permuted channel is reconstructed by inverse bit packing, as given in Equation (26):

(26) $${\tilde{C}}^{\left(c\right)}=B^{-1}\left({\tilde{B}}^{\left(c\right)}\right)$$

The final permuted RGB image is obtained as shown in Equation (27):

(27) $$\tilde{I}=\left({\tilde{C}}^{\left(R\right)},{\tilde{C}}^{\left(G\right)},{\tilde{C}}^{\left(B\right)}\right)$$

with distinct traversal types assigned to each channel. Since $\pi_{m_{c}}$ is bijective, the permutation stage is fully invertible and introduces no information loss.

### 2.6. Encryption and Decryption Algorithms

In Section 2.1, Section 2.2, Section 2.3, Section 2.4 and Section 2.5, the structural mechanisms and algorithms used to encode and decode data are described in two algorithms. The architecture of the encryption algorithm is based on GAN-based key generation, PWLCM-derived dual diffusion, complementary operations at the nucleotide level, and channel-specific zigzag bit-plane permutation. The data are recovered by following the same operational pathway in reverse order of the encoding process. Because all transformations are inherently reversible, the system enables complete and accurate reconstruction of the original visual data.

The encryption process is initiated by a single image-specific noise vector (as described in Algorithms 1 and 2). To decrypt the encrypted image, two components are required: the correct noise vector and the trained generator model. If either of these components is missing or incorrect, the original image cannot be reconstructed, which enhances the overall security of the cryptosystem.
**Algorithm 1. Encryption Process****Input:** Plain image *P* (H × W × 3), Trained generator *G*, Noise dimension *d* = 100 **Output:** Cipher image C, Stored noise vector z 1: Sample *z* ~ *N* (0, 1) ∈ ℜ*^d^* 2: *K* ← *G*(z)        // Generate 256 × 256 × 3 key image via trained GAN 3: Store z          // Only the compact noise vector is preserved 4: x ← Flatten(K) [1:100]/255 // Extract first 100 elements 5: Compute *A, B, C, D* from x using Equation (6) 6: Normalize A, B, C, D to [0.1, 0.9] using Equation (7) 7: N←H×W×3 8: $Q^{\left(1\right)}\leftarrow PWLCM(\hat{A},\hat{B},N+1,discard=100)$ 9: $S_{1}\leftarrow \left\lfloor 256Q_{1:N}^{\left(1\right)}\right\rfloor ,IV_{f}\leftarrow \left\lfloor 256Q_{N+1}^{\left(1\right)}\right\rfloor$ 10: $Q^{\left(2\right)}\leftarrow PWLCM(\hat{C},\hat{D},N,discard=100)$ 11: $S_{2}\leftarrow Reshape\left(\left\lfloor 256Q^{\left(2\right)}\right\rfloor ,H,W,3\right)$ 12: X1 ← P ⊕ K      // GAN-based initial mixing 13: $M\leftarrow Flatten(X_{1})\;$ // Interleaved RGB byte sequence 14: $F\leftarrow ForwardDiffuse(M,S_{1},IV_{f})$ // Forward chained diffusion 15: $X_{2}\leftarrow Reshape(F,H,W,3)$ 16: **for** each channel *c* ∈ {R, G, B} **do** 17:        **for** each pixel (*i, j*) **do** 18:                 *X*_2_*^c^*(*i, j*) ← DNAComplement(*X*_2_*^c^*(*i, j*)) 19:        **end for** 20: **end for** 21: methods ← [serpentine, spiral, mirrored diagonal] 22: **for** each channel *c* ∈ {R, G, B} **do** 23:        *B^c^* ← BitExpand(*X_2_^c^*) 24:        *B^c^* ← Permute(*B^c^*, methods[*c*]) 25:    **if**
c=R **then** 26:      $B^{c}\leftarrow ShiftHighBitRows(B^{c},3)$ 27:    **end if** 28:        *Z_c_ ←BitPack(*$B^{c}$*)* 29: **end for** 30: Z ← Merge(Z_R_, Z_G_, Z_B_) 31: K_r_ ← S_2_ ⊕K 32: K_c_ ← S_2_ 33: RowIVs, ColumnIVs ← DeriveIVs(K, SHA-256, Equation (14)) 34: R^b^ ← BidirectionalRowDiffuse(Z,K_r_ ,RowIVs) 35: C ← BidirectionalColumnDiffuse(R^b^,K_c_ ,ColumnIVs) 36: **return**
*C*, *z*


**Algorithm 2. Decryption Process**
**Input:** Cipher image *C*, Stored noise vector *z*, Trained generator *G* **Output:** Recovered plain image *P*′ 1: *K* ← *G*(z)        // *Regenerate identical key image from stored noise* 2: x ← Flatten(K) [1:100]/255 3: Compute *A, B, C, D* from *x* using Equation (6) 4: Normalize A, B, C, D to [0.1, 0.9] using Equation (7) 5: N←H×W×3 6: $Q^{\left(1\right)}\leftarrow PWLCM(\hat{A},\hat{B},N+1,discard=100)$ 7: $S_{1}\leftarrow \left\lfloor 256Q_{1:N}^{\left(1\right)}\right\rfloor ,IV_{f}\leftarrow \left\lfloor 256Q_{N+1}^{\left(1\right)}\right\rfloor$ 8: $Q^{\left(2\right)}\leftarrow PWLCM(\hat{C},\hat{D},N,discard=100)$ 9: $S_{2}\leftarrow Reshape\left(\left\lfloor 256Q^{\left(2\right)}\right\rfloor ,H,W,3\right)$ 10: K_r_ ← S_2_ ⊕K 11: K_c_ ← S_2_ 12: RowIVs, ColumnIVs ← DeriveIVs(K, SHA-256, Equation (14)) 13: R^b^ ← InverseColumnDiffuse (C, K_c_, ColumnIVs) 14: Z ← InverseRowDiffuse (R^b^, K_r_, RowIVs) 15: methods ← [serpentine, spiral, mirrored diagonal] 16: **for** each channel *c* ∈ {R, G, B} **do** 17:        *B^c^* ← BitExpand(Z_c_) 18:    **if**
c=R **then** 19:      $B^{c}\leftarrow \;$InverseShiftHighBitRows$\left(B^{c},3\right)$ 20:    **end if** 21:       *B^c^ ← InversePermute(B^c^, methods[c])* 22:       *X*_2_*^c^*   ←BitPack*(*$B^{c}$
*)* 23: **end for** 24: *X_2_ ← Merge(X_2,R_, X_2,G_, X_2,B_)* 25: **for** each channel c∈{R,G,B} **do** 26:    **for** each pixel $\left(i,j\right)$ **do** 27:      $X_{2,c}(i,j)\leftarrow DNAComplement(X_{2,c}(i,j))$ 28:    **end for** 29: **end for** 30: $F\leftarrow Flatten(X_{2})$ 31: $M\leftarrow InverseForwardDiffuse(F,S_{1},IV_{f})$ 32: $X_{1}\leftarrow Reshape(M,H,W,3)$ 33: $P’\leftarrow X_{1}⊕K$34: **return**
*P*′

## 3. Experimental Results and Performance Metrics

A rigorous set of criteria is used to evaluate the cryptographic strength of the proposed algorithm. Each metric provides an objective basis for assessing encryption quality, the randomness of the encrypted data, and the overall robustness of the algorithm against various types of cryptanalytic attacks.

### 3.1. Histogram Analysis

Pixel intensity distributions can be analyzed using histograms. Generally, plain images contain uneven pixel value distributions; however, a good encryption system should have a uniform (flat) histogram for its cipher image [43,44]. Uniformity indicates that all pixel values have been sufficiently randomized and that no meaningful statistical information remains for use against the cryptosystem in statistical analysis [45,46].

**Figure 6:**
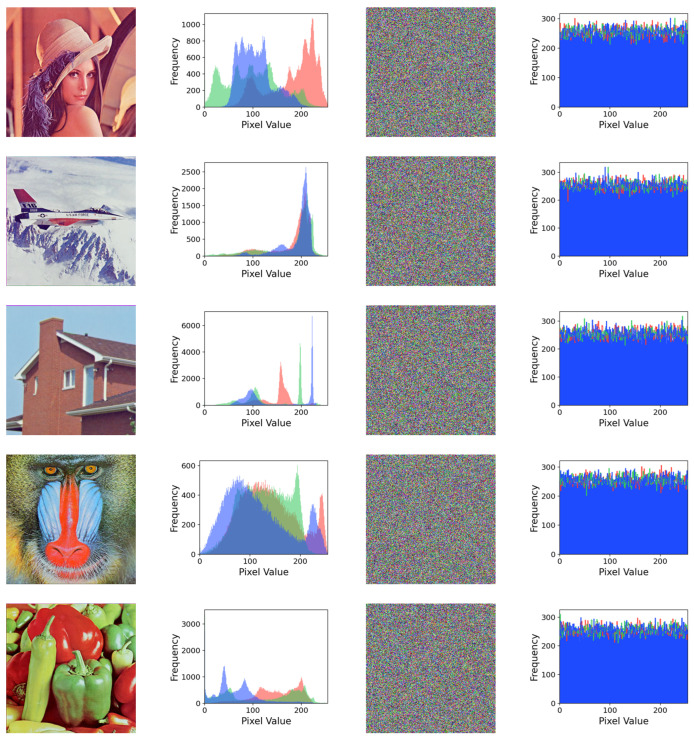

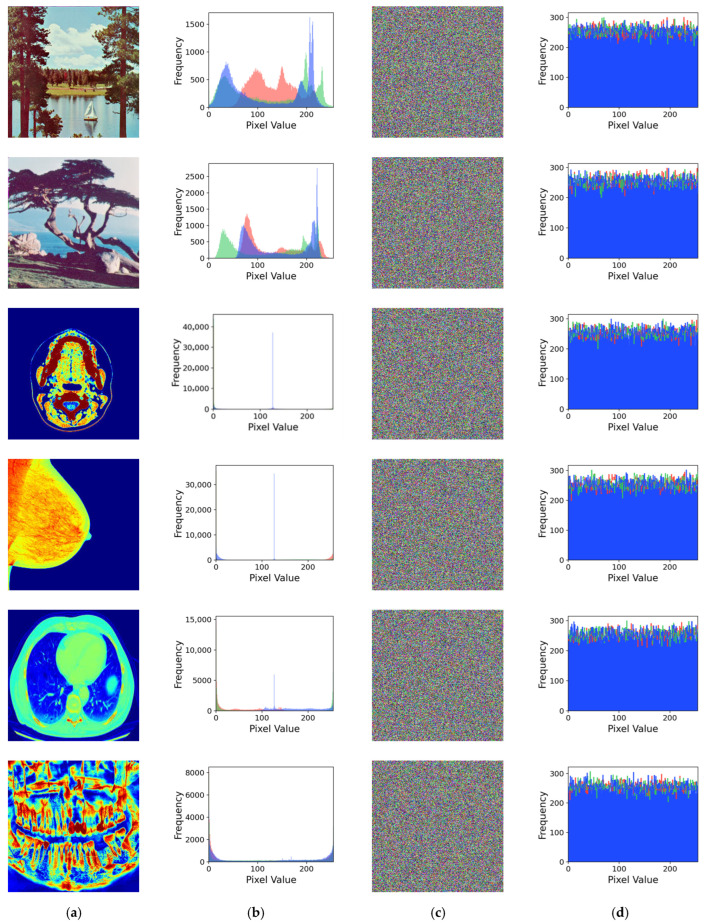
Histogram analysis of plain and encrypted test images: (**a**) plain images, (**b**) histograms of plain images, (**c**) encrypted images, and (**d**) histograms of encrypted images.

The histogram evaluations of eight test images can be seen in Figure 6; they are arranged as (a) the plain image, (b) the histogram of the plain image, (c) the corresponding cipher image, and (d) the histogram of the cipher image. A strong level of randomization is evident in the pixel intensity distribution of the cipher image when compared with its corresponding plain image, indicating effective masking of the statistical characteristics of the original plaintext image, which demonstrates that this encryption system has strong resistance to statistical attacks.

### 3.2. Quality Measurements (MSE, PSNR, SSIM)

PSNR has been widely used as a standard metric to measure reconstruction accuracy and visual similarity between images. In cryptographic evaluations, PSNR is interpreted differently, as it can also be used to quantify the spatial divergence between plaintext and ciphertext. Extremely low PSNR represents a high degree of structural deviation, indicating that the ciphering mechanism has effectively obscured all visual information from the images being compared.

The Peak Signal-to-Noise Ratio (PSNR) is defined as given in Equation (28):

(28) $$PSNR=20\cdot \log_{10}\left(\frac{MAX}{\sqrt{MSE}}\right)$$

In this formulation, MAX denotes the maximum pixel intensity, equal to 255 for standard 8-bit images. To systematically measure the average squared error between the original and transformed pixel values, the Mean Squared Error (MSE) is defined as follows in Equation (29):

(29) $$MSE=\frac{\sum {\left(y_{i}-p_{i}\right)}^{2}}{n}$$

In this formulation, $y_{i}$ and $p_{i}$ correspond to the intensity levels of the $i^{th}$ pixel in the original and processed images, respectively, while n defines the total number of pixels (M × N). Analytically, a Mean Squared Error (MSE) approaching zero indicates near-perfect structural similarity, while higher MSE values reflect significant visual divergence between the plaintext and the encrypted image.

The Structural Similarity Index Measure evaluates image quality using luminance, contrast, and structure [47], rather than relying solely on conventional pixel-level error measurements. For cryptographic applications, a significantly low SSIM value indicates substantial structural divergence between the plaintext and the cipher image; minimal similarity indicates that the cryptosystem effectively disrupts spatial structures and obscures the original visual content [48].

The SSIM is formulated as given in Equation (30):

(30) $$SSIM\left(x,y\right)=\frac{\left(2\mu_{x}\mu_{y}+a_{1}\right)\left(2\sigma_{xy}+a_{2}\right)}{\left(\mu_{x}^{2}+\mu_{y}^{2}+a_{1}\right)\left(\sigma_{x}^{2}+\sigma_{y}^{2}+a_{2}\right)}$$

Within this mathematical algorithm, $\mu_{x}$ and $\mu_{y}$ define the mean luminance values of images x and y, respectively, whereas $\sigma_{x}^{2}$ and $\sigma_{y}^{2}$ represent their corresponding variances, capturing the spread of pixel values around these means. To quantify the structural relationship between corresponding pixels, the formulation incorporates the covariance term $\sigma_{xy}$. The variables $a_{1}$ and $a_{2}$ function as small stabilizing constants introduced to prevent division-by-zero errors and maintain numerical stability during computation. Ultimately, the SSIM index is bounded between −1 and +1, where values approaching +1 indicate greater structural similarity between the compared images.

Table 3 summarizes the MSE, PSNR, and SSIM results of the proposed encryption algorithm for all test images over the RGB channels. The algorithm achieves high MSE values, low PSNR values, and near-zero SSIM values, indicating a strong visual divergence between the plain and encrypted images. The MSE values range between 6.8119 × 10^3^ for the House-R channel and 20.3962 × 10^3^ for the Breast-R channel. Similarly, the PSNR values remain below 10 dB for all tested images, ranging from 5.0353 dB for the Breast-R channel to 9.7981 dB for the House-R channel. These results indicate that the proposed encryption algorithm effectively conceals the visual content of the original images.

Across all channels, the SSIM values range from −0.0003 to 0.0133. The negative SSIM value obtained for the Brain-R channel indicates the absence of structural similarity between the plain and cipher images. In addition, the average SSIM values of all test images remain close to zero and do not exceed 0.0098, confirming that the proposed algorithm effectively disrupts inherent structural correlations.

For the medical images, Brain, Breast, Chest, and Dental produce consistently high average MSE values of 15.7502 × 10^3^, 16.4165 × 10^3^, 15.0622 × 10^3^, and 16.1955 × 10^3^, respectively. Their corresponding average PSNR values remain low, with 6.4586 dB, 6.1045 dB, 6.4958 dB, and 6.0381 dB, while the average SSIM values are close to zero, ranging from 0.0027 to 0.0059. These results show that the proposed method maintains a consistent encryption effect across different medical image types. This indicates that the algorithm is not limited to a single medical image structure and can effectively obscure visual and structural information in medical images with different characteristics.

The MSE, PSNR, and SSIM values of the proposed algorithm and existing encryption techniques for the Lena image are compared in Table 4. The proposed algorithm obtains an MSE value of 9.0176 × 10^3^ and a PSNR value of 8.6398 dB. In terms of PSNR, the proposed algorithm remains within the same range as the compared methods and is slightly higher than the values reported by Cemile et al. [49], Pal et al. [50], Alexan et al. [51], and Aqeel et al. [52], while remaining very close to the result of Alexan et al. [53]. The MSE value is close to those reported by Pal et al. [50], Alexan et al. [51], and Alexan et al. [53], indicating that the proposed method produces a comparable level of pixel-level distortion between the plain and encrypted Lena images.

Although most of the compared studies do not report SSIM values, the proposed algorithm provides an SSIM value of 0.0087. This near-zero SSIM value indicates that the encrypted Lena image preserves almost no structural similarity with the corresponding plain image. Therefore, the comparative results show that the proposed algorithm achieves a competitive distortion level while effectively disrupting structural information in the encrypted image.

### 3.3. Entropy Analysis

Information entropy is considered an important statistical measure for determining the randomness of visual data. It provides an objective value that can be used to quantify the predictability of encrypted data, depending on the bit-depth of the system. In the case of an 8-bit system, it is well established that the theoretical maximum is 8, as noted in [54,55]. The closer the entropy of the cipher approaches this theoretical maximum, the more uniform the pixel intensity distribution becomes. Therefore, a highly random pixel intensity distribution significantly reduces correlations between adjacent pixels, providing the cryptosystem with resistance to statistical analysis and differential cryptanalysis [56].

The information entropy is defined as given in Equation (31):

(31) $$H=-\sum_{i=1}^{N}p_{i}*\log_{2}\left(p_{i}\right)$$

where N is defined as the total number of possible pixel intensity levels (256 for 8-bit images), and $p_{i}\;$corresponds to the probability of the $i^{th}$ intensity level occurring in the image.

According to design principles for an 8-bit grayscale representation, the maximum possible information entropy can reach a value of 8. As entropy approaches this theoretical limit, the pixel intensity distribution becomes more uniform. Thus, such a uniformly distributed representation makes it difficult for an attacker to obtain significant statistical information and perform statistical attacks. In comparison, a plain image usually has much lower entropy values because it tends to contain many spatial correlations [57].

Table 5 contains the detailed characterization of the encrypted images with respect to information entropy measurements. All encrypted images exceed 7.996 across all RGB channels, and their entropy values approach the theoretical maximum value of 8 within a very narrow range, indicating a near-uniform distribution of pixel intensities. In contrast, the entropy values of the corresponding plain images vary considerably, ranging from 3.0889 for the Brain-G channel to 7.7526 for the Mandrill-B channel.

For the medical images, the original average entropy values differ substantially according to image content and structural complexity. The Brain and Breast images have relatively low average entropy values of 3.6189 and 3.7546, respectively, whereas the Chest and Dental images produce higher average entropy values of 6.0307 and 6.7890. Despite these differences, their corresponding encrypted images achieve highly similar average entropy values of 7.9972, 7.9972, 7.9973, and 7.9973, respectively. This indicates that the proposed algorithm can produce a near-uniform intensity distribution for medical images with different entropy characteristics.

Overall, despite the variation in the entropy values of the plain images, all cipher images exhibit highly consistent entropy values within the range of 7.9967–7.9976. Additionally, the variation across RGB channels remains minimal, approximately ±0.0005. Achieving such high entropy values indicates that the encryption algorithm effectively obscures the underlying visual information and provides resistance against statistical cryptanalysis.

The analysis of information entropy per channel for the Lena image, presented in Table 6, confirms that the proposed algorithm exhibits strong randomness characteristics due to the high entropy values obtained in all RGB channels (R = 7.9972, G = 7.9975, and B = 7.9968). These values are very close to the theoretical maximum value of 8, indicating that the encrypted Lena image has a near-uniform pixel distribution.

Compared with the existing methods listed in Table 6, the proposed algorithm provides entropy values within the same high-randomness range as the compared encryption algorithms. The proposed method achieves a higher red-channel entropy than Neamah and Shukur [58] and Zhang and Wang [60], while matching the red-channel result reported by Alexan et al. [53]. In the green channel, the proposed method yields the highest entropy value among the compared methods. In the blue channel, although some studies report slightly higher values, the proposed result remains very close to them and stays within the same high-entropy range.

Overall, the entropy values obtained by the proposed algorithm approach the ideal value and demonstrate competitive randomness performance compared with the existing encryption algorithms. The small variation among the RGB channels further indicates that the proposed method maintains a balanced randomness distribution across color components and effectively reduces statistical information leakage in the encrypted image.

### 3.4. Differential Attack Analysis

To determine how sensitive an encryption algorithm is to plaintext attacks, differential analysis is performed on the encryption process using statistical measures known as the Number of Pixels Change Rate (NPCR) and the Unified Average Changing Intensity (UACI). The NPCR calculates the proportion of pixels that change between two cipher images generated from plaintexts differing by a single-pixel modification. In parallel, the UACI measures the mean intensity difference between these ciphertexts. Optimal cryptographic performance is typically associated with theoretical values of approximately 99.6094% for NPCR and 33.4635% for UACI [61,62]. In this study, the NPCR and UACI values are computed under a strict differential protocol in which the secret key is held fixed and only the plaintext is perturbed. Specifically, the image-specific noise vector z and the trained generator G remain identical for both encryptions, and consequently so do the GAN-generated key image K = G(z), the derived PWLCM control parameters, the chaotic key stream S_1_, the chaotic matrix S_2_, and all row and column diffusion materials. The two plaintexts P_1_ and P_2_ differ only in the red-channel value at the pixel with zero-based coordinates (128,128), corresponding to pixel (129,129) under one-based indexing; the value is incremented by one modulo 256 for each test image, and for the Lena image this corresponds to a change from 161 to 162, while no key-dependent quantity is altered between the two runs. The resulting non-zero NPCR and UACI values therefore quantify plaintext diffusion under a fixed key rather than key sensitivity, which is reported separately in Section 3.7.

The NPCR is defined as given in Equation (32):

(32) $$NPCR=\frac{\sum_{i,j}D\left(i,j\right)}{M\times N}\times 100\%$$

The UACI is defined as given in Equation (33):

(33) $$UACI=\frac{1}{M\times N}\sum_{i,j}\frac{\left|C_{1}\left(i,j\right)-C_{2}\left(i,j\right)\right|}{255}\times 100\%$$

Contextualizing Equations (32) and (33), the variables $C_{1}(i,j)$ and $C_{2}(i,j)$ represent the corresponding pixel intensity values at location $\left(i,j\right)$ in two encrypted outputs generated from plaintexts differing by a single pixel, both encrypted under identical key material (the same image-specific noise vector z and trained generator G); thus, the only difference between the two encryption instances is the single-pixel change introduced in the plaintext, while all key-derived quantities remain unchanged. The binary matrix D(i,j) is assigned a value of 1 when the compared pixel values differ $C_{1}(i,j)\neq C_{2}(i,j)$ and 0 otherwise, where M×N denotes the total number of pixels within the image.

Table 7 quantifies the NPCR and UACI characteristics of the proposed algorithm across the entire test dataset’s RGB channels. The recorded NPCR measurements range between 99.2996% for the Breast-B channel and 99.8413% for the Airplane-R channel. Although certain channel-wise NPCR values deviate from the theoretical reference value of 99.6094%, these deviations appear in isolated channels rather than as a repeated or systematic pattern across the dataset. In particular, the Lena image produces an average NPCR value of 99.6028%, which is nearly identical to the theoretical reference, while the remaining images also remain within a close band around this value. Therefore, the observed channel-level fluctuations should be interpreted as content-dependent variations rather than as evidence of a structural weakness in the proposed encryption process.

The UACI values also remain close to the theoretical expected value of 33.4635%, ranging from 32.7983% for the Mandrill-B channel to 34.0084% for the Chest-R channel. For the Lena image, the average UACI value is 33.4634%, showing almost exact agreement with the theoretical reference. Although some individual channel values are slightly above or below the ideal value, the average UACI results consistently stay near the expected level. This confirms that the intensity variation caused by a one-pixel modification is effectively transferred to the encrypted image.

For the medical images, Brain, Breast, Chest, and Dental produce average UACI values of 33.3726%, 33.5441%, 33.5395%, and 33.5525%, respectively. Their corresponding average NPCR values are 99.6526%, 99.5143%, 99.6485%, and 99.5021%. These results indicate that the proposed method preserves high differential sensitivity across different medical image types despite their distinct structural properties. Although the Breast and Dental images produce slightly lower average NPCR values than the theoretical reference, their corresponding UACI values remain close to the ideal level, supporting that the effect of a one-pixel plaintext change is still distributed effectively throughout the cipher image.

Overall, the NPCR and UACI results confirm that the proposed algorithm provides strong resistance against differential attacks. The small variations observed in some individual RGB channels do not form a persistent or image-independent pattern, and therefore do not indicate a consistent vulnerability. On the contrary, the average results, particularly those obtained for the Lena image, remain in very close agreement with the theoretical expectations and support the differential robustness of the proposed scheme.

In Table 8, the results of the proposed algorithm are presented in comparison with existing encryption techniques based on the computed differential metrics for the Lena image. The proposed algorithm achieves an NPCR value of 99.60% and a UACI value of 33.46%, both of which are very close to the theoretical reference values of 99.6094% and 33.4635%, respectively. This indicates that the proposed method produces a differential response that is well aligned with the expected behavior of a secure image encryption scheme under a one-pixel plaintext modification.

Compared with the methods listed in Table 8, the proposed algorithm remains within the same ideal differential range as the strongest reported studies. Although some methods report slightly higher NPCR values, such as Pal et al. [50] and Neamah and Shukur [58], and Cao and Song [64] report a rounded NPCR value of 99.61%, the result of the proposed method stays very close to the theoretical reference. Therefore, from the perspective of theoretical proximity, the proposed NPCR result can be considered fully competitive with the compared methods.

In terms of UACI, the proposed algorithm achieves a value of 33.46%, which is almost identical to the theoretical expected value and consistent with several of the strongest reported results in the table. While Cao and Song [64] report 33.47% and Pal et al. [50] report 33.52%, the differences remain very small. By contrast, the lower UACI values reported by Sharma et al. [65], Alexan et al. [51], and Meng and Wu [59] indicate a comparatively weaker average intensity change under a one-pixel modification.

Overall, the comparative results demonstrate that the proposed algorithm provides competitive differential attack resistance. Its NPCR and UACI values are both closely aligned with the theoretical references and remain fully comparable to the results of the existing encryption algorithms listed in Table 8.

### 3.5. Correlation Analysis

The linear relationship between adjacent horizontal, vertical, and diagonal pixels is typically measured using correlation analysis. It can be observed that unaltered images show a very large amount of structural similarity and high spatial correlation among neighboring pixel groups. A secure encryption algorithm should cause this correlation in the cipher image to approach zero. The degree to which this correlation is reduced indicates that spatial correlation has been effectively reduced; hence, it becomes difficult for an attacker to obtain meaningful statistical information. The correlation coefficient is defined as given in Equation (34):

(34) $$r_{xy}=\frac{\sum_{i=1}^{N}\left(x_{i}-\bar{x}\right)\left(y_{i}-\bar{y}\right)}{\sqrt{\sum_{i=1}^{N}{\left(x_{i}-\bar{x}\right)}^{2}\sum_{i=1}^{N}{\left(y_{i}-\bar{y}\right)}^{2}}}$$

Contextualizing the equation, $x_{i}$ and $y_{i}$ represent the intensity values of adjacent pixels across horizontal, vertical, or diagonal directions. Evaluating these values relative to their corresponding sequence means ($\bar{x}$ and $\bar{y}$) over N randomly selected pixel pairs yields the correlation coefficient $r_{xy}$. Bounded within the interval [−1, 1], this coefficient quantifies spatial correlation, where values approaching 0 indicate weak pixel dependency and values approaching ±1 indicate strong spatial correlation.

**Figure 7:**
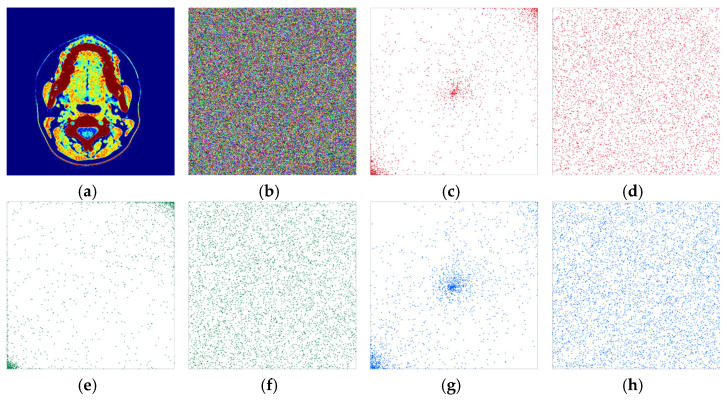
Horizontal adjacent pixel correlation scatter plots for the Brain test image: (**a**) plain image, (**b**) encrypted image, (**c**) red channel scatter plot of the plain image, (**d**) red channel scatter plot of the encrypted image, (**e**) green channel scatter plot of the plain image, (**f**) green channel scatter plot of the encrypted image, (**g**) blue channel scatter plot of the plain image, and (**h**) blue channel scatter plot of the encrypted image.

The horizontal correlation scatter plots of the Brain image’s RGB channels (Figure 7) show a high density of pixels in a diagonal arrangement within the plaintext, indicating a very high amount of spatial dependence. However, the distribution of pixels in the scatter plots for the ciphertext is much more uniform than the distribution of the pixels in the plaintext; thus, there is essentially a completely uniform distribution of pixels on the scatter plots of the ciphertext (indicating that the encryption algorithm has effectively removed any remaining spatial dependencies between adjacent pixels).

The correlation coefficients in all spatial directions, as shown in Table 9, indicate that a high degree of similarity exists in the plaintext images, with values ranging from 0.69 to 0.99. This correlation is significantly reduced after encryption, with most coefficients falling within the interval [−0.0085, 0.0076]. Only a few coefficients exceed |0.008| in absolute value, such as Tree-B diagonal (−0.0085) and Sailboat on lake-R horizontal (−0.0084), yet their magnitudes remain very small. Therefore, the algorithm effectively reduces statistical dependencies between adjacent pixels, as evidenced by the near-random distribution of correlation coefficients in the encrypted images.

Table 9 presents the horizontal, vertical, and diagonal correlation coefficients and provides an initial range of plaintext dependencies from 0.6995 (Mandrill-G, diagonal) to 0.9962 (Breast-R, horizontal). The encryption technique significantly reduces these internal dependencies to near-zero values in all spatial directions. This transformation is clearly observed in the highly structured House image (horizontal: 0.9765, vertical: 0.9525, diagonal: 0.9357), which yields encrypted correlation values of −0.0019, 0.0024, and 0.0042, respectively.

The reduction in spatial dependencies is further demonstrated by the decrease in the Mandrill diagonal correlation from 0.7891 to −0.0023. Minor deviations in certain values, such as Tree-B diagonal (−0.0085) and Sailboat on lake-R horizontal (−0.0084), remain minimal. Overall, the predominance of near-zero correlation values indicates that the encryption algorithm significantly reduces inherent spatial dependencies and provides resistance against statistical attacks.

A similar behavior is observed for the medical images. In particular, the Brain, Breast, Chest, and Dental images exhibit high adjacent-pixel correlations in their plain forms, whereas their encrypted versions produce correlation coefficients close to zero in the horizontal, vertical, and diagonal directions. This indicates that the proposed method effectively suppresses spatial dependencies across medical images with different structural characteristics.

Table 10 presents a comparative analysis of multidirectional correlation coefficients for the Lena image, where the proposed algorithm records values of 0.0046, 0.0007, and 0.0023 in the horizontal, vertical, and diagonal directions, respectively. These values are very close to zero, indicating that the encrypted image has very weak adjacent-pixel dependency in all directions.

Compared with the existing methods listed in Table 10, the proposed algorithm remains within the same near-zero correlation range as the competing methods. In the vertical direction, the proposed method provides one of the lowest absolute correlation values and performs better than Man and Song [63], Shakhmetova et al. [66], Cemile et al. [49], Alexan et al. [51], and Zhang and Wang [60], while Cao and Song [64] report a slightly lower absolute value. In the horizontal direction, the proposed method achieves a lower absolute correlation value than Shakhmetova et al. [66], Cemile et al. [49], Alexan et al. [51], and Zhang and Wang [60], although Man and Song [63] and Cao and Song [64] report slightly smaller values. In the diagonal direction, the proposed method performs better than Cemile et al. [49], Alexan et al. [51], and Zhang and Wang [60], while remaining very close to the low-correlation values reported by the other compared studies.

Overall, the proposed algorithm achieves near-zero correlation coefficients in all three directions and shows competitive decorrelation performance compared with the existing encryption algorithms. These results support that the proposed method effectively suppresses adjacent-pixel dependencies and strengthens resistance against statistical attacks.

### 3.6. Key Space Analysis

For a cryptographic encryption method to be secure, there must be a sufficiently large number of possible keys to prevent brute-force attacks. This is achieved by having a large key space and, therefore, high key complexity [67]. According to current cryptographic standards, the minimum effective key space is $2^{128}$, while $2^{256}$ is recommended to ensure long-term security (e.g., according to NIST).

A distinctive property of the proposed cryptosystem is that the GAN-generated key image K, the four PWLCM control parameters $\left(\hat{A},\hat{B},\hat{C},\hat{D}\right)$, the first chaotic key stream and its initial value ($S_{1}$, $IV_{f}$), and the second chaotic RGB matrix $S_{2}$ are deterministically generated from the 100-dimensional noise vector $z\in R^{100}$ through the protected trained generator G. Thus, none of these components contributes independent entropy. For a fixed trained generator, the per-image key space is determined by the representation of the vector z.

All elements of z are independently sampled and represented using double-precision 64-bit floating-point format (IEEE 754). Under this representation, each dimension provides approximately $2^{52}$ distinguishable states due to the precision of the mantissa. Therefore, the effective key space can be estimated as:

(35) $$\left|K\right|\;\approx \;2^{5200}\;\approx \;10^{1565}$$

For this per-image key-space estimation, the protected trained generator G is assumed to remain fixed, and only the search space of the image-specific noise vector is considered. Specifically, each dimension is assumed to be represented with a conservative practical precision of $10^{-14}$. Under this assumption, the effective key space is reduced to approximately $10^{1400}$, which is equivalent to about $2^{4650}$.

Even with this conservative estimation, the resulting key space remains several orders of magnitude larger than the recommended security threshold of $2^{128}$ and significantly exceeds that of AES-256, indicating a nominal search space that is computationally prohibitive for exhaustive enumeration.

Furthermore, successful decryption requires both the exact image-specific noise vector and the original trained GAN generator. Possession of the noise vector alone is therefore insufficient to regenerate the byte-identical key image and the associated chaotic diffusion materials.

It is acknowledged that the theoretical key space of 2^5200^, derived from the IEEE 754 double-precision representation of the 100-dimensional latent vector, should be interpreted only as an upper bound rather than as the exact effective key space. In practice, the effective number of distinguishable GAN-generated key images may be smaller because a trained generator may map different latent vectors to identical or perceptually similar outputs. This limitation is consistent with the well-known mode-collapse problem in GANs, where the generator may produce samples with limited diversity or cover only a subset of the target distribution [68,69]. Similar concerns have also been reported in the security analysis of deep-learning-based image cryptosystems. In particular, Wang et al. analyzed a GAN-based DeepKeyGen medical image cryptosystem and showed that low sensitivity in key design and generation may lead to practical security vulnerabilities; their experiments reported low NPCR and UACI values under minor input changes, indicating insufficient sensitivity to plaintext variations [70].

To mitigate this limitation, the proposed scheme does not rely solely on the GAN-generated key image. Instead, the GAN output is used as one component within a multi-layer encryption structure reinforced by dual PWLCM-based chaotic diffusion, DNA complement transformation, and channel-specific bit-level permutation. This design is consistent with recent hybrid GAN–chaos image encryption frameworks, where GAN-based encryption is combined with chaotic scrambling and diffusion mechanisms to address insufficient key space, weak key-parameter sensitivity, and low encryption complexity [71]. The empirical key sensitivity analysis in Section 3.7 further supports this argument: a single-bit modification in the noise vector produces highly different cipher images, with NPCR values exceeding 99.4% across all tested channels. Therefore, the $2^{5200}$ value is reported only as a theoretical upper-bound estimate and should not be interpreted as a proven effective key-space size. Because the trained generator maps latent vectors to generated key images, the actual effective key space may be lower due to output redundancy, learned-manifold constraints, or mode-collapse-related effects. Accordingly, no precise numerical lower bound is claimed for the effective GAN-derived key space. The nominal latent-vector search space is nevertheless substantially larger than the $2^{256}$ key space of AES-256 [72], while the practical resistance of the proposed cryptosystem is further evaluated through the key sensitivity and differential-security analyses reported in Section 3.7.

### 3.7. Key Sensitivity Analysis

Key sensitivity analysis is a fundamental metric for the security evaluation of image encryption algorithms. This metric is designed to measure the difference in output between two cipher images as a result of slight changes (i.e., changes of one or more bits) in the secret key, especially with respect to the robustness of the encryption algorithm. A well-designed encryption algorithm should exhibit this property, rendering it computationally infeasible for an attacker to derive the original key by exploring nearby key values in the key space through one-bit modifications in the secret key [73,74].

The NPCR outcomes derived from the key sensitivity analysis for Lena and Brain images across the RGB channels are summarized in Table 11. In all cases, the NPCR values exceed 99.59% for each channel, suggesting that even a slight alteration in the secret key, such as a single-bit change, produces substantial variations in the encrypted images. This behavior indicates that the proposed encryption algorithm possesses a high level of key sensitivity and provides strong resistance against differential attacks.

**Figure 8:**
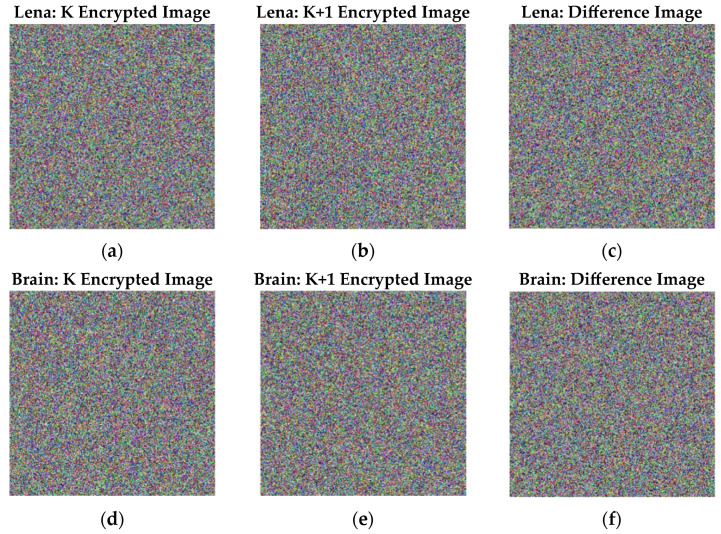
Visual Results of Key Sensitivity Analysis. (**a**) Lena encrypted image using the original key K; (**b**) Lena encrypted image using the modified key K+1; (**c**) difference image between (**a**) and (**b**); (**d**) Brain encrypted image using the original key K; (**e**) Brain encrypted image using the modified key K+1; (**f**) difference image between (**d**) and (**e**).

Figure 8 demonstrates the key sensitivity characteristics of the proposed method. Subfigures (a) and (d) correspond to the encrypted images generated using the original key K, whereas subfigures (b) and (e) present the results obtained with a slightly modified key (i.e., K + 1-bit change). Subfigures (c) and (f) show the associated difference images. It can be observed that even a minor key variation produces entirely uncorrelated cipher images, and the resulting difference images display a noise-like random pattern. These findings confirm that the proposed algorithm ensures strong key sensitivity and eliminates any statistical dependency between encrypted outputs produced by closely related keys.

### 3.8. Known-Plaintext and Chosen-Plaintext Attack Analysis

Adversaries conducting chosen-plaintext attacks (CPAs) use entirely black or entirely white images as input to exploit weaknesses in the encryption algorithm and compromise system integrity. The ability to encrypt completely homogeneous images evaluates the structural resistance of the proposed algorithm toward targeted plaintext manipulation [75].

**Figure 9:**
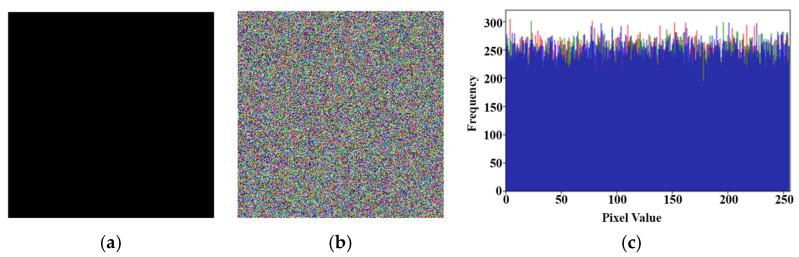

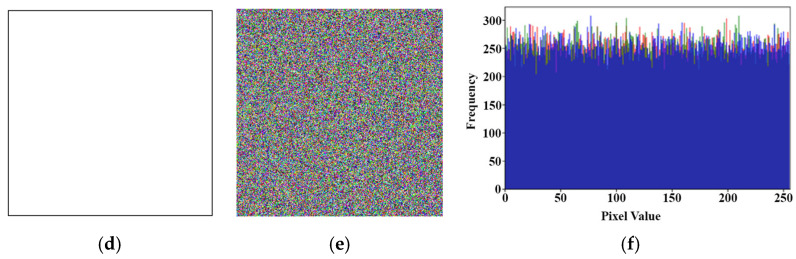
Chosen-plaintext attack (CPA) analysis results: (**a**) entirely black plain image, (**b**) encrypted image of (**a**), (**c**) histogram of (**b**), (**d**) entirely white plain image, (**e**) encrypted image of (**d**), and (**f**) histogram of (**e**). Red, green, and blue lines in the histograms correspond to the respective color channels of the RGB image.

The cipher images and their corresponding histograms, as shown in Figure 9, exhibit high randomness and uniform pixel distributions compared to the original images. The proposed encryption algorithm reduces potential statistical leakage from plaintexts with maximum spatial homogeneity. However, since the homogeneous-image test alone does not address keystream-recovery attacks, the following analysis examines the structural resistance of the proposed scheme to known-plaintext and chosen-plaintext attacks that attempt to extract reusable keystream material.

A known-plaintext or chosen-plaintext attack against an additive stream-cipher structure typically attempts to recover the keystream from a plaintext-ciphertext pair and then reuse it to decrypt other ciphertexts produced under the same key. The specific cross-instance equivalent-keystream reuse attack raised in the review applies when the encryption can be reduced to a reusable plaintext-independent mapping such as C = P ⊕ keystream, in which the same keystream remains reusable across encryptions. The proposed algorithm does not satisfy this condition.

First, the encryption process is plaintext dependent. After the initial XOR with the GAN-generated key image, the forward chained diffusion stage computes each output byte by combining the current masked plaintext byte with the corresponding chaotic driving value and the previously generated output byte. Therefore, each encrypted byte depends not only on its own input value but also, recursively, on the preceding plaintext content. This dependency is further propagated by the subsequent row and column diffusion stages, which spread the influence of local changes along both spatial directions. As a consequence, the effective transformation at each position cannot be expressed as a fixed additive keystream that is independent of the plaintext.

Second, the keying material is image specific. For each encryption instance, a newly sampled noise vector z is used to generate the key image K = G(z), and all subsequent diffusion materials, including the PWLCM control parameters, the forward diffusion stream, the initial diffusion value, and the row and column diffusion keys, are derived from K. Consequently, even if the key material associated with one plaintext-ciphertext pair were fully recovered, it would remain valid only for that specific encryption instance and would provide no reusable keystream for decrypting another ciphertext generated from a different z. Consequently, keystream material recovered from one ciphertext cannot be reused to decrypt ciphertexts generated under a different noise vector, unlike in plaintext-independent stream-cipher constructions.

Within the threat model defined in Section 2.1, reproducing the key image K and the associated diffusion materials further requires both the image-specific noise vector z and the protected generator G. The observed cipher images alone do not provide sufficient information to reproduce these materials: z is transmitted only through a protected channel and is never embedded in the ciphertext, while reconstructing the generator G would require access to its learned parameters, which are not revealed by the cipher images. Therefore, beyond the statistical-leakage resistance demonstrated by the homogeneous-image experiment, the preceding structural analysis indicates that key material associated with one encryption instance cannot be reused against ciphertexts generated using a different noise vector.

### 3.9. Cropping and Noise Attack Analysis

Applying simulated data loss and transmission errors to encrypted outputs through cropping and noise attacks allows effective evaluation of the robustness of the encryption protocol’s structural stability. To quantify this resiliency, the present study truncates the encrypted data output at predetermined levels of 10%, 25%, 50%, and 75% [76,77].

Table 12 illustrates how progressive cropping of the ciphertext enables comparison between truncated ciphertexts and recovered outputs. As data is cropped from 10% to 75% of the original ciphertext, there is a reduction in clarity during decryption; however, the core visual content remains recognizable even at 75% data loss. Thus, the consistent recovery of the images indicates that the algorithm effectively distributes pixel information across the entire encrypted image, reducing the risk of localized data loss.

Table 13 illustrates how the system responds to targeted noise interference using salt-and-pepper corruption at density levels of 10%, 25%, and 50%. Even with such significant modifications to the data, the recovered images each exhibit distinct and recognizable primary visual characteristics within all three test levels. The consistent clarity of the decrypted images strongly supports the proposed encryption system’s significant structural robustness against cropping and noise manipulation.

### 3.10. Computational Complexity and Encryption Speed

To evaluate the practical applicability of the proposed algorithm, a runtime benchmark was conducted on a 256 × 256 RGB test image using the hardware and software environment described in Section 2.1. The benchmark excludes model loading and disk I/O, measuring only the online encryption path including latent vector generation, GAN inference, parameter derivation, PWLCM-based diffusion material generation, GAN XOR masking, forward diffusion, DNA complement transformation, zigzag permutation, and row/column diffusion.

The mean total encryption time was 1876.12 ms per image, yielding a throughput of 0.533 images per second (0.035 MPix/s). As presented in Table 14, a breakdown of the processing stages reveals that the zigzag permutation accounts for the largest portion of the total runtime (986.06 ms, 52.56%), followed by DNA complement transformation (275.03 ms, 14.66%), row diffusion (186.01 ms, 9.91%), column diffusion (181.12 ms, 9.65%), forward diffusion (120.10 ms, 6.40%), and PWLCM diffusion material generation (97.70 ms, 5.21%). The GAN pipeline contributes 27.90 ms (1.49%) of the total runtime, while parameter derivation and GAN XOR masking collectively account for only a negligible fraction of the overall cost. These results indicate that the dominant computational burden arises from permutation and diffusion operations rather than from the GAN-based key generation stage.

The benchmark was conducted in a CPU-only environment due to runtime constraints, yielding a throughput of 0.533 images/s (0.035 MPix/s). In GPU-accelerated environments, the encryption pipeline is expected to achieve significantly higher throughput. Therefore, the reported runtime represents a conservative upper bound, and real-world performance in GPU-enabled clinical environments is anticipated to be substantially faster.

### 3.11. Ablation Study

To quantify the individual contribution of each cryptographic layer, a systematic ablation study was conducted by progressively adding components to the encryption pipeline. Table 15 reports cumulative ablation results; that is, each row corresponds to the ciphertext produced after inserting the indicated component into the pipeline and evaluating the resulting partial encryption chain.

When the GAN-generated key image is applied alone without any subsequent diffusion or permutation, the encrypted output exhibits high residual spatial correlation, with horizontal, vertical, and diagonal correlation coefficients of 0.5842, 0.6144, and 0.5516, respectively. The entropy value of 7.7711 and the extremely low NPCR value of 0.0005 further confirm that the GAN layer alone is insufficient for secure encryption. This result is consistent with the design intent of the proposed system, in which the GAN component serves primarily as a key-image generation and per-image key-management component rather than as a standalone encryption primitive.

The addition of PWLCM-based chaotic diffusion produces the most significant improvement in the statistical behavior of the cipher image. Horizontal, vertical, and diagonal correlation coefficients drop to 0.0028, 0.0057, and 0.0016, respectively, while the entropy rises to 7.9969. At the same time, the NPCR value increases sharply to 86.6699, indicating that the PWLCM-driven diffusion layers constitute the primary source of confusion and diffusion in the proposed system.

The DNA complement transformation further improves the encryption characteristics by introducing additional bit-level nonlinearity. With the inclusion of the DNA layer, the entropy increases slightly to 7.9972, while the horizontal, vertical, and diagonal correlation coefficients are reduced further to 0.0003, −0.0006, and −0.0010, respectively. In the cumulative ablation setting reported in Table 15, the NPCR value increases from 86.6699 to 98.8820 when the DNA layer is inserted before the subsequent row and column diffusion stages. Therefore, this increase should be interpreted as the combined effect of the DNA-domain remapping and the downstream chained diffusion, rather than as an isolated effect of the DNA complement operation alone.

Finally, inserting the channel-specific zigzag permutation before the existing row and column diffusion stages increases the NPCR value to 99.6028, which is very close to the theoretical reference. The full pipeline also preserves near-zero correlation coefficients in all spatial directions, with horizontal, vertical, and diagonal values of 0.0036, 0.0007, and 0.0023, respectively, while maintaining an entropy value of 7.9972. These results confirm the complementary nature of the encryption layers and validate the necessity of each component within the proposed multi-layer architecture.

These results further indicate that the GAN-generated image should be interpreted as reproducible key material rather than as a standalone encryption output. Accordingly, the final cryptographic performance is primarily determined by the subsequent chaotic diffusion, DNA-level transformation, and bit-level permutation stages built upon that key material.

## 4. Conclusions

This research proposes the integration of a multi-layer encryption algorithm for medical images that combines the following components: a Generative Adversarial Network (GAN) for key generation, multi-stage chained diffusion based on the Piecewise Linear Chaotic Map (PWLCM), DNA complement transformation, and channel-specific zigzag bit-plane permutation. These components together create an end-to-end algorithm in which an image-specific 100-dimensional noise vector is used to generate the key image, while the chaotic control parameters and diffusion materials are derived from the generated key image. Therefore, no full-size key image storage is required. Since the noise vector alone is insufficient to regenerate the key image without the trained generator, its transmission alone does not expose the encryption key, thereby reducing per-image key storage and transmission overhead under a pre-shared protected-generator assumption.

To evaluate the proposed medical image encryption algorithm, eleven images, including medical images and standard benchmark test images were used to perform multiple evaluation tests. In every case, the resulting cipher images exhibit uniformly distributed pixel intensities and significant structural degradation (i.e., high Mean Squared Error (MSE), very low Peak Signal-to-Noise Ratio (PSNR, below 10 dB), and very low Structural Similarity Index (SSIM) values near zero). Thus, the original images are almost completely obscured, with minimal visual characteristics remaining. Across all RGB channels of every test image, including the extended set of medical images, the cipher outputs exhibit very high spatial randomness (entropy values greater than 7.996), approaching the theoretical maximum of 8. Even for the Brain medical image, which contains large homogeneous regions, the average entropy value remains high at 7.9972, indicating consistent behavior across different image types.

The differential attack analysis results similarly indicate the effectiveness of the method, achieving an NPCR of 99.60% and a UACI of 33.46% for the Lena image, which are consistent with theoretical expectations for secure encryption. Correlation analysis further confirms that pixel dependencies are reduced to within [−0.01, 0.01] across all spatial directions. In addition, the proposed method transforms highly homogeneous inputs (entirely black and white images) into cipher outputs with uniform statistical properties, demonstrating reduced statistical leakage for highly homogeneous plaintexts.

The ablation analysis further confirms the complementary contribution of each encryption layer. The dual PWLCM-based diffusion stage provides the dominant improvement in statistical randomness and correlation reduction, while the DNA complement transformation introduces an additional reversible bit-level nonlinear operation. The final channel-specific zigzag permutation further reduces residual spatial dependency and completes the multi-layer confusion structure.

Finally, even under substantial degradation of the cipher image (e.g., 75% cropping and 50% salt-and-pepper noise), the decrypted images preserve the primary visual content of the original images at a recognizable level, demonstrating the robustness of the proposed algorithm against data loss and noise interference. Comparative analysis with existing methods demonstrates that the proposed approach achieves competitive performance across all evaluation metrics, while offering the additional advantage of a compact per-image key-management mechanism under a pre-shared protected-generator model. Successful decryption requires: (1) the identical image-specific 100-dimensional noise vector and (2) the original trained GAN generator. As such, possession of either component alone is insufficient for successful decryption.

Although the proposed method demonstrates strong robustness, the current implementation is restricted to 256 × 256 images due to the fixed input dimension of the trained GAN generator. The computational analysis also shows that the proposed method can be executed in a CPU-only environment for 256 × 256 RGB images, while identifying the channel-specific zigzag permutation as the dominant computational component rather than the GAN inference stage. Future work will focus on extending the algorithm to support larger image resolutions and optimizing computational efficiency, particularly for real-time deployment in medical imaging applications such as Magnetic Resonance Imaging (MRI), Computed Tomography (CT), Ultrasound, and DICOM-based systems.

## Figures and Tables

**Table 1:** DNA Encoding and Complementary Mapping Rule.

Original Bit Pair	DNA Representation	DNA Complement	Encoded Bit Pair
00	A	T	01
01	T	A	00
10	C	G	11
11	G	C	10

**Table 2:** Bit-level DNA encoding of a sample RGB pixel. Red, green, and blue highlights correspond to the R, G, and B color channels, respectively.

Pixel Position (i, j)	**R(0,0)**								**G(0,0)**								**B(0,0)**							
Original Pixel Value	173								92								150							
Original Binary Value	1	0	1	0	1	1	0	1	0	1	0	1	1	1	0	0	1	0	0	1	0	1	1	0
DNA Representation	C		C		G		T		T		T		G		A		C		T		T		C	
Encoded DNA Output	G		G		C		A		A		A		C		T		G		A		A		G	
Encoded Binary Value	1	1	1	1	1	0	0	0	0	0	0	0	1	0	0	1	1	1	0	0	0	0	1	1
Encoded Pixel Value	248								9								195							

**Table 3:** MSE, PSNR, and SSIM values of the encrypted images for each RGB channel across the test dataset.

Image	Channel	MSE (×10^3^)	PSNR (dB)	SSIM
Airplane	R	10.1557	8.0637	0.0085
	G	10.8763	7.7660	0.0076
	B	10.5097	7.9149	0.0079
	Average	10.5139	7.9149	0.0080
Brain	R	18.7958	5.3902	−0.0003
	G	20.0183	5.1165	0.0012
	B	8.4365	8.8692	0.0074
	Average	15.7502	6.4586	0.0027
Breast	R	20.3962	5.0353	0.0032
	G	17.4861	5.7039	0.0056
	B	11.3671	7.5743	0.0062
	Average	16.4165	6.1045	0.0050
Chest	R	15.1457	6.3279	0.0048
	G	19.6403	5.1993	0.0037
	B	10.4005	7.9602	0.0091
	Average	15.0622	6.4958	0.0059
Dental	R	16.7345	5.8947	0.0052
	G	15.9004	6.1167	0.0051
	B	15.9514	6.1028	0.0028
	Average	16.1955	6.0381	0.0044
House	R	6.8119	9.7981	0.0095
	G	8.6576	8.7568	0.0086
	B	9.6330	8.2932	0.0108
	Average	8.3675	8.9494	0.0096
Lena	R	10.7040	7.8354	0.0110
	G	9.2196	8.4837	0.0061
	B	7.1292	9.6004	0.0090
	Average	9.0176	8.6398	0.0087
Mandrill	R	8.5133	8.8298	0.0090
	G	7.7005	9.2656	0.0052
	B	9.4011	8.3990	0.0094
	Average	8.5383	8.8315	0.0079
Peppers	R	8.0328	9.0821	0.0096
	G	11.3009	7.5997	0.0080
	B	11.2544	7.6176	0.0053
	Average	10.1960	8.0998	0.0076
Sailboat on lake	R	7.2830	9.5077	0.0103
	G	11.6675	7.4610	0.0105
	B	11.7172	7.4426	0.0085
	Average	10.2226	8.1371	0.0098
Tree	R	8.6509	8.7602	0.0133
	G	11.3526	7.5798	0.0045
	B	9.6970	8.2644	0.0101
	Average	9.9001	8.2015	0.0093

**Table 4:** Comparative analysis of MSE, PSNR, and SSIM values between the proposed method and existing encryption algorithms.

	MSE (×10^3^)	PSNR (dB)	SSIM
Proposed	9.0176	8.6398	0.0087
Cemile et al. [49]	N/A	8.6358	N/A
Pal et al. [50]	9.0230	8.5645	N/A
Alexan et al. [51]	8.9198	8.6272	N/A
Aqeel et al. [52]	N/A	7.8463	N/A
Alexan et al. [53]	8.9837	8.6510	N/A

**Table 5:** Information entropy values of plain and encrypted images for each RGB channel across the test dataset.

Image	Channel	Original Image	Encrypted Image
Airplane	R	6.8567	7.9969
	G	6.9564	7.9973
	B	6.3351	7.9972
	Average	6.7161	7.9971
Brain	R	3.6695	7.9975
	G	3.0889	7.9971
	B	4.0981	7.9971
	Average	3.6189	7.9972
Breast	R	3.4264	7.9972
	G	4.2907	7.9968
	B	3.5466	7.9977
	Average	3.7546	7.9972
Chest	R	6.0809	7.9975
	G	4.8763	7.9973
	B	7.1350	7.9971
	Average	6.0307	7.9973
Dental	R	6.6483	7.9971
	G	6.6698	7.9973
	B	7.0490	7.9975
	Average	6.7890	7.9973
House	R	6.4311	7.9967
	G	6.5389	7.9974
	B	6.2320	7.9970
	Average	6.4007	7.9970
Lena	R	7.2992	7.9972
	G	7.6453	7.9975
	B	6.9985	7.9968
	Average	7.3143	7.9972
Mandrill	R	7.6756	7.9974
	G	7.4793	7.9969
	B	7.7526	7.9976
	Average	7.6358	7.9973
Peppers	R	7.3449	7.9972
	G	7.5607	7.9974
	B	7.1003	7.9973
	Average	7.3353	7.9973
Sailboat on lake	R	7.3207	7.9969
	G	7.6720	7.9975
	B	7.3042	7.9968
	Average	7.4323	7.9971
Tree	R	7.2104	7.9973
	G	7.4136	7.9971
	B	6.9207	7.9971
	Average	7.1816	7.9972

**Table 6:** Comparative analysis of information entropy values between the proposed method and existing encryption algorithms.

	Red (R)	Green (G)	Blue (B)
Proposed Method	7.9972	7.9975	7.9968
Aqeel et al. [52]	7.9973	7.9970	7.9974
Neamah & Shukur [58]	7.9969	7.9972	7.9974
Alexan et al. [53]	7.9972	7.9965	7.9963
Meng & Wu [59]	7.9973	7.9974	7.9974
Zhang & Wang [60]	7.9971	7.9969	7.9978

**Table 7:** NPCR (%) and UACI (%) values of the encrypted images for each RGB channel across the test dataset.

	Channel	UACI	NPCR
Airplane	R	33.7900	99.8413
	G	33.5191	99.6048
	B	33.7308	99.7955
	Average	33.6800	99.7472
Brain	R	33.4100	99.6017
	G	33.5227	99.5758
	B	33.1850	99.7803
	Average	33.3726	99.6526
Breast	R	33.9587	99.6231
	G	33.3441	99.6201
	B	33.3295	99.2996
	Average	33.5441	99.5143
Chest	R	34.0084	99.7986
	G	33.4118	99.5605
	B	33.1982	99.5865
	Average	33.5395	99.6485
Dental	R	33.5582	99.4232
	G	33.4521	99.6063
	B	33.6472	99.4766
	Average	33.5525	99.5021
House	R	33.6907	99.4217
	G	33.5902	99.6078
	B	33.3344	99.5987
	Average	33.5384	99.5427
Lena	R	33.1566	99.4186
	G	33.4906	99.6033
	B	33.7430	99.7864
	Average	33.4634	99.6028
Mandrill	R	33.5659	99.4202
	G	33.3965	99.5911
	B	32.7983	99.6002
	Average	33.2536	99.5372
Peppers	R	33.4398	99.8032
	G	33.7516	99.6017
	B	33.1709	99.7650
	Average	33.4541	99.7233
Sailboat on lake	R	32.9559	99.4446
	G	33.3617	99.6307
	B	32.9740	99.7650
	Average	33.0972	99.6134
Tree	R	32.7989	99.5773
	G	33.4320	99.6078
	B	33.6249	99.7696
	Average	33.2853	99.6516

**Table 8:** Comparative analysis of NPCR and UACI values between the proposed method and existing encryption algorithms.

	NPCR	UACI
Proposed Method	99.60	33.46
Man & Song [63]	99.62	33.46
Cao & Song [64]	99.61	33.47
Sharma et al. [65]	99.42	32.03
Cemile et al. [49]	99.61	33.46
Pal et al. [50]	99.63	33.52
Alexan et al. [51]	99.61	30.39
Aqeel et al. [52]	99.61	33.45
Neamah & Shukur [58]	99.64	33.46
Meng & Wu [59]	99.60	33.41

**Table 9:** Correlation coefficients of adjacent pixels in horizontal, vertical, and diagonal directions for plain and encrypted images across each RGB channel.

		Original Image			Encrypted Image		
Image	Channel	Horizontal	Vertical	Diagonal	Horizontal	Vertical	Diagonal
Airplane	R	0.9109	0.8930	0.8271	0.0045	0.0018	0.0006
	G	0.8997	0.9058	0.8363	−0.0009	−0.0003	−0.0006
	B	0.9259	0.8731	0.8357	0.0004	0.0001	−0.0046
	Average	0.9122	0.8906	0.8330	0.0013	0.0005	−0.0015
Brain	R	0.9100	0.9261	0.8721	−0.0043	−0.0004	−0.0008
	G	0.8935	0.9165	0.8568	−0.0010	0.0004	0.0041
	B	0.8099	0.8426	0.7393	−0.0030	−0.0041	−0.0033
	Average	0.8711	0.8951	0.8227	−0.0028	−0.0014	0.0000
Breast	R	0.9962	0.9960	0.9938	0.0013	−0.0049	0.0035
	G	0.9846	0.9817	0.9763	0.0019	0.0007	0.0013
	B	0.9798	0.9818	0.9717	−0.0025	−0.0021	−0.0024
	Average	0.9869	0.9865	0.9806	0.0003	−0.0021	0.0008
Chest	R	0.9431	0.9572	0.9177	0.0007	0.0020	−0.0070
	G	0.9735	0.9823	0.9593	0.0047	−0.0021	−0.0064
	B	0.9039	0.9105	0.8467	−0.0028	−0.0024	−0.0028
	Average	0.9402	0.9500	0.9079	0.0009	−0.0008	−0.0054
Dental	R	0.8613	0.9181	0.8252	−0.0018	−0.0030	−0.0071
	G	0.7974	0.8751	0.7407	0.0068	−0.0050	0.0024
	B	0.8616	0.9164	0.8244	−0.0063	−0.0037	−0.0008
	Average	0.8401	0.9032	0.7968	−0.0004	−0.0039	−0.0018
House	R	0.9671	0.9353	0.9126	−0.0053	0.0014	0.0026
	G	0.9805	0.9474	0.9320	0.0012	0.0045	0.0025
	B	0.9820	0.9749	0.9625	−0.0015	0.0012	0.0076
	Average	0.9765	0.9525	0.9357	−0.0019	0.0024	0.0042
Lena	R	0.9379	0.9662	0.9058	0.0042	0.0023	0.0016
	G	0.9157	0.9531	0.8818	0.0023	−0.0026	0.0048
	B	0.8921	0.9283	0.8522	0.0043	0.0022	0.0004
	Average	0.9152	0.9492	0.8799	0.0036	0.0007	0.0023
Mandrill	R	0.9132	0.8737	0.8522	0.0073	−0.0003	−0.0037
	G	0.8024	0.7557	0.6995	−0.0008	0.0071	−0.0035
	B	0.8772	0.8647	0.8156	−0.0046	0.0002	0.0003
	Average	0.8643	0.8314	0.7891	0.0006	0.0023	−0.0023
Peppers	R	0.9494	0.9534	0.9129	−0.0067	0.0016	−0.0010
	G	0.9593	0.9651	0.9299	−0.0038	0.0031	−0.0009
	B	0.9408	0.9496	0.9033	−0.0032	0.0039	−0.0010
	Average	0.9498	0.9560	0.9154	−0.0046	0.0029	−0.0010
Sailboat on lake	R	0.9330	0.9304	0.8959	−0.0084	−0.0031	0.0017
	G	0.9339	0.9293	0.8898	0.0018	−0.0044	0.0016
	B	0.9407	0.9465	0.9098	0.0012	−0.0073	0.0071
	Average	0.9359	0.9354	0.8985	−0.0018	−0.0049	0.0035
Tree	R	0.9590	0.9361	0.9159	−0.0023	−0.0050	−0.0023
	G	0.9687	0.9457	0.9318	−0.0004	−0.0020	−0.0043
	B	0.9612	0.9406	0.9265	0.0021	0.0043	−0.0085
	Average	0.9630	0.9408	0.9247	−0.0002	−0.0009	−0.0050

**Table 10:** Comparative analysis of correlation coefficients between the proposed method and existing encryption algorithms.

	Horizontal	Vertical	Diagonal
Proposed	0.0046	0.0007	−0.0023
Man & Song [63]	0.0028	0.0019	−0.0011
Cao & Song [64]	−0.0026	−0.0003	0.0022
Shakhmetova et al. [66]	−0.0066	−0.0106	−0.0014
Cemile et al. [49]	0.0142	0.0197	−0.0099
Alexan et al. [51]	0.0079	−0.0015	−0.0036
Zhang & Wang [60]	0.0050	−0.0021	0.0034

**Table 11:** NPCR Results for Key Sensitivity Analysis.

	Red	Green	Blue
Lena (256 × 256)	99.6307	99.6262	99.5728
Brain (256 × 256)	99.6658	99.6262	99.6277

**Table 12:** Cropping attack resilience analysis: (a)–(d) encrypted images with 10%, 25%, 50%, and 75% cropping rates; (e)–(h) corresponding decrypted images.

	10% Crop	25% Crop	50% Crop	75% Crop
*Cropped*	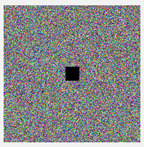	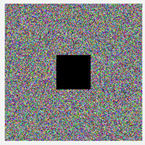	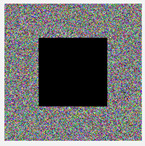	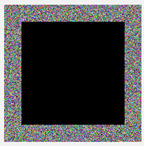
	(a)	(b)	(c)	(d)
*Decrypted*	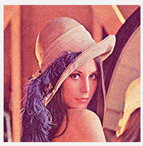	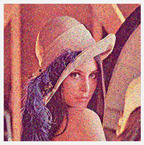	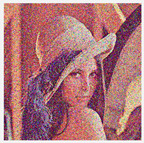	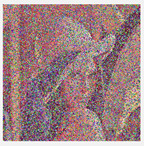
	(e)	(f)	(g)	(h)

**Table 13:** Salt-and-pepper noise attack resilience analysis: (a)–(c) encrypted images corrupted with 10%, 25%, and 50% noise levels; (d)–(f) corresponding decrypted images.

	10% Noise	25% Noise	50% Noise
*Noisy*	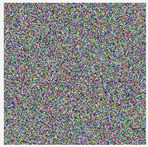	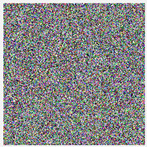	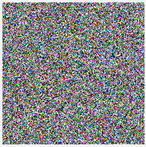
	(a)	(b)	(c)
*Decrypted*	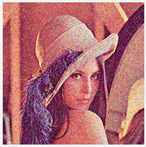	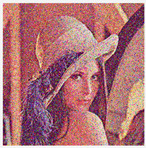	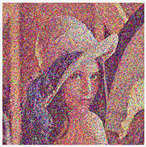
	(d)	(e)	(f)

**Table 14:** Encryption runtime breakdown for a 256 × 256 RGB image.

	Time (ms)	Percentage (%)
GAN pipeline	27.90	1.49
Parameter derivation (GAN → A, B, C, D)	1.72	0.09
PWLCM diffusion material generation	97.70	5.21
GAN XOR masking	0.45	0.02
Forward diffusion	120.10	6.40
DNA complement transformation	275.03	14.66
Zigzag permutation	986.06	52.56
Row diffusion	186.01	9.91
Column diffusion	181.12	9.65
Total	1876.12	100.00

**Table 15:** Ablation study results analysis of the encryption.

	Entropy	Horizontal	Vertical	Diagonal	NPCR
GAN only	7.7711	0.5842	0.6144	0.5516	0.0005
GAN + PWLCM	7.9969	0.0028	0.0057	0.0016	86.6699
GAN + PWLCM + DNA	7.9972	0.0003	−0.0006	0.0010	98.8820
Full Encryption Algorithm	7.9972	0.0036	0.0007	0.0023	99.6028

## Data Availability

The Acute Lymphoblastic Leukemia (ALL) dataset used for GAN training is publicly available on Kaggle (https://www.kaggle.com/datasets/mehradaria/leukemia, accessed on 4 March 2026). Medical Imaging (CT-Xray) Colorization New Dataset used as part of the experimental evaluation is publicly available on Kaggle (https://www.kaggle.com/datasets/shuvokumarbasak2030/medical-imaging-ct-xray-colorization-new-dataset, accessed on 4 March 2026). The remaining standard test images used in the experimental evaluation are publicly available benchmark images.

## References

[1] Masood F., Driss M., Boulila W., Ahmad J., Rehman S.U., Jan S.U., Qayyum A., Buchanan W.J. (2021). A Lightweight Chaos-Based Medical Image Encryption Scheme Using Random Shuffling and XOR Operations. Wirel. Pers. Commun..

[2] Wang X., Tu C. (2020). A Chaos-Based Medical Image Encryption Method. Indones. J. Electr. Eng. Comput. Sci..

[3] El-Shafai W., Khallaf F., El-Rabaie E.S., El-Samie F.E. (2022). Proposed 3D Chaos-Based Medical Image Cryptosystem for Secure Cloud-IoMT eHealth Communication Services. J. Ambient Intell. Humaniz. Comput..

[4] Amarouche S., Talbi S., Daoui M., Couchot J.F. (2023). 2D-ZasHen Chaotic Map-Based Medical Image Cryptographic Approach in IoT-Based Healthcare Monitoring. Proceedings of IEEE/ACS International Conference on Computer Systems and Applications, AICCSA.

[5] Rhouma R., Arroyo D., Belghith S. (2009). A New Color Image Cryptosystem Based on a Piecewise Linear Chaotic Map. International Multi-Conference on Systems, Signals & Devices.

[6] Chen Y., Tang C., Yi Z. (2020). A Novel Image Encryption Scheme Based on PWLCM and Standard Map. Complexity.

[7] Dib S., Benchiheb A., Benmeddour F. (2022). Robust Chaos-Based Medical Image Cryptosystem. Wseas Trans. Commun..

[8] Zhang Z., Zhang J. (2024). Parallel Multi-Image Encryption Based on Cross-Plane DNA Manipulation and a Novel 2D Chaotic System. Vis. Comput..

[9] Ding P., Geng P., Hu W. (2024). A New Controllable Multi-Wing Chaotic System: Applications in High-Security Color Image Encryption. J. Supercomput..

[10] Huang Y.H., Zhang Q.L., Zhao Y.B. (2025). Color Image Encryption Algorithm Based on Hybrid Chaos and Layered Strategies. J. Inf. Secur. Appl..

[11] Mathivanan P., Maran P. (2023). Color Image Encryption Based on Novel Kolam Scrambling and Modified 2D Logistic Cascade Map (2D LCM). J. Supercomput..

[12] Huang Y., Huang H., Huang Y., Wang Y., Yu F., Yu B., Liu C. (2024). Asymptotic Shape Synchronization in Three-Dimensional Chaotic Systems and Its Application in Color Image Encryption. Chaos Solitons Fractals.

[13] Zhang H., Hu H., Ding W. (2024). VSDHS-CIEA: Color Image Encryption Algorithm Based on Novel Variable-Structure Discrete Hyperchaotic System and Cross-Plane Confusion Strategy. Inf. Sci..

[14] Wang X., Wu H., Yan A., Hao H. (2026). Construction of Multi-State Chaotic Systems and Applications to Image Encryption. Sci. Rep..

[15] Vargas Valencia J.A., Londoño-Arboleda M.A., Salinas Jiménez H.D., Marín Arango C.A., Duque Gómez L.F. (2026). Image Encryption Using Chaotic Box Partition–Permutation and Modular Diffusion with PBKDF2 Key Derivation. J. Cybersecur. Priv..

[16] Wang X.Y., Zhang H.L., Bao X.M. (2016). Color Image Encryption Scheme Using CML and DNA Sequence Operations. BioSystems.

[17] Alghafis A., Firdousi F., Khan M., Batool S.I., Amin M. (2020). An Efficient Image Encryption Scheme Based on Chaotic and Deoxyribonucleic Acid Sequencing. Math. Comput. Simul..

[18] Liu X., Wang P., Tang D., Tian J. (2024). A New Four-Dimensional Memristive System, Synchronization and Its Application in Image Encryption. Int. J. Dyn. Control.

[19] Li R., Liu T., Yin J. (2024). An Encryption Algorithm for Color Images Based on an Improved Dual-Chaotic System Combined with DNA Encoding. Sci. Rep..

[20] Dash S., Padhy S., Kumar N., Nayyar A. (2026). Medisecure: A Hybrid Approach for Enhancing Multimedia Data Protection in Healthcare. Clust. Comput..

[21] Al-Shargabi B., Al-Husainy M.A.F., Abuarqoub A., Aldabbas O.A. (2026). A Robust Image Encryption Method Based on the Randomness Properties of DNA Nucleotides. Comput. Mater. Contin..

[22] Wang Q., Yang Y., Zhang X. (2026). Real-Time Medical Image Encryption Algorithm Based on Punch Scrambling and Fast Hachimoji DNA Coding. Expert Syst. Appl..

[23] Tang J., Lin J., Hong Y., Hou J., Zhao Y. (2026). A Novel Chaotic Image Encryption Scheme Based on Block Compressive Sensing. Nonlinear Dyn..

[24] Cheng M., Jiang M., Yang J., Zhan C., Wang X., Lin F., Liang W. (2026). Chaotic Multi-Image Encryption Scheme Based on New Spatial Permutation and BP Neural Network Compression. Nonlinear Dyn..

[25] Ding Y., Tan F., Qin Z., Cao M., Choo K.K.R., Qin Z. (2022). DeepKeyGen: A Deep Learning-Based Stream Cipher Generator for Medical Image Encryption and Decryption. IEEE Trans. Neural Netw. Learn. Syst..

[26] Duggirala S.K., Sathya M. (2026). A Hybrid Deep Learning Framework Utilizing CNN, GAN, and Autoencoder for Safe Medical Image Encryption in IoMT. Eng. Res. Express.

[27] Afzal S., Bokhari M.U., Luqman M., Hanafi B. (2026). Lightweight CNN-Based Edge Computing with Hybrid Chaotic Encryption for Secure and Efficient Image Transmission in IoT Networks. Discov. Comput..

[28] Wu Y., Liu X., Cascone L., Nappi M., Wan S. (2025). Plausible Deniable Medical Image Encryption by Large Language Models and Reversible Content-Aware Strategy. IEEE J. Biomed. Health Inform..

[29] Wang M., Guo Z., Yan X. (2026). Facial Image Encryption Algorithm Based on Feature Recognition and Chaotic Scrambling. J. Inf. Secur. Appl..

[30] Gopalakrishnan K., Ahilan A., Deepa P., Muthukumaran N. (2026). SCAM-MODEL: Secure Cardiac MRI Image Encryption via Lightweight Pixel-Permutation-Based Horsy Chess Model. Int. J. Comput. Intell. Syst..

[31] Wang Z., Xu N., Qi D., Zhang S. (2026). Multi-Image Optical Encryption Based on Physics-Enhanced Deep Neural Network. Opt. Laser Technol..

[32] Podder D., Deb S., Banik D., Kar N., Sahu A.K. (2024). Robust Medical and Color Image Cryptosystem Using Array Index and Chaotic S-Box. Clust. Comput..

[33] Kumar A., Dua M. (2024). A Novel Exponent–Sine–Cosine Chaos Map-Based Multiple-Image Encryption Technique. Multimed. Syst..

[34] Ullah S., Liu X., Waheed A., Zhang S. (2025). S-Box Using Fractional-Order 4D Hyperchaotic System and Its Application to RSA Cryptosystem-Based Color Image Encryption. Comput. Stand. Interfaces.

[35] Safdar M.U., Shah T., Ali A. (2024). Design of Nonlinear Component of Block Cipher over Non-Chain Semi-Local Ring with Its Application to Color Image Encryption. Arab. J. Sci. Eng..

[36] Inam S., Kanwal S., Niazi M., Karamti H., Al-Otaibi S., Jamjoom M.M. (2026). A Novel Hybrid Image Encryption Scheme Using Henon Map for Secure Image Communication. IET Inf. Secur..

[37] Wu N., Chen Z., Zheng Z., Yao M. (2026). Cross-Channel Color Image Encryption Scheme Based on a Novel 3D Hyperchaotic Map, Peano Curve and Compressed Sensing. Phys. Scr..

[38] Liu L., Huang B., Lai S. (2026). Multi-Image Encryption Based on an Extended Chaotic Map and a Directional Shift Transformation. Chaos Solitons Fractals.

[39] Amiri S., Zaied M. (2026). DeepCryptanalysis: Dense Attention U-Net to Break Chaos-Based Color Image Encryption. Adv. Artif. Intell. Mach. Learn..

[40] Onakpojeruo E.P., Mustapha M.T., Ozsahin D.U., Ozsahin I. (2024). A Comparative Analysis of the Novel Conditional Deep Convolutional Neural Network Model, Using Conditional Deep Convolutional Generative Adversarial Network-Generated Synthetic and Augmented Brain Tumor Datasets for Image Classification. Brain Sci..

[41] Zhang J., Wen H. (2024). Dynamic Feedback Bit-Level Image Privacy Protection Based on Chaos and Information Hiding. Sci. Rep..

[42] Shahna K.U., Mohamed A. (2020). A Novel Image Encryption Scheme Using Both Pixel Level and Bit Level Permutation with Chaotic Map. Appl. Soft Comput..

[43] Chowdhary C.L., Patel P.V., Kathrotia K.J., Attique M., Kumaresan P., Ijaz M.F. (2020). Analytical Study of Hybrid Techniques for Image Encryption and Decryption. Sensors.

[44] Qiu H., Zhang X., Yue H., Liu J. (2023). A Novel Eighth-Order Hyperchaotic System and Its Application in Image Encryption. Mathematics.

[45] Sharma P., Shrivastava R., Sarthi V.K., Bhatpahri P. (2018). Security Analysis of XOR Based Ciphered Image. Asian J. Comput. Sci. Technol..

[46] Sivakumar P., Yusoff Y. (2024). Comparative Study on Encryption Algorithms for Autism MRI Scan Images. Int. J. Innov. Comput..

[47] Wang Z., Bovik A.C., Sheikh H.R., Simoncelli E.P. (2004). Image Quality Assessment: From Error Visibility to Structural Similarity. IEEE Trans. Image Process..

[48] Hussein N.M., Mohammed N.M. (2023). Using the Chaotic to Improve the RC4 Algorithm. Int. Res. J. Innov. Eng. Technol..

[49] İnce C., İnce K., Hanbay D. (2024). Novel Image Pixel Scrambling Technique for Efficient Color Image Encryption in Resource-Constrained IoT Devices. Multimed. Tools Appl..

[50] Pal S., Mahanty A., Pathak A., Karmakar J., Mondal H., Mandal M.K. (2023). A Novel Image Encryption Technique with Four Stage Bit-Interspersing and A 4D-Hyperchaotic System. ECTI-CIT Trans..

[51] Alexan W., Hosny K., Gabr M. (2025). A New Fast Multiple Color Image Encryption Algorithm. Clust. Comput..

[52] Aqeel M., Jaffar A., Faheem M., Ashraf M.W., Iqbal N., Yousaf S., Diab H. (2025). A Randomized Non-Overlapping Encryption Scheme for Enhanced Image Security in Internet of Things (IoT) Applications. Eng. Rep..

[53] Alexan W., Elkandoz M., Mashaly M., Azab E., Aboshousha A. (2023). Color Image Encryption Through Chaos and KAA Map. IEEE Access.

[54] Harjo B., Setiadi D.R.I.M. (2021). Improved Color Image Encryption Using Hybrid Modulus Substitution Cipher and Chaotic Method. Int. J. Intell. Eng. Syst..

[55] Liu P., Ouyang J., Shao Z., Kumar Pal P., Kumar D. (2024). Zirili Map-Based Image Encryption Method for Healthcare, Military, and Personal Data Security. Phys. Scr..

[56] Arab A., Rostami M.J., Ghavami B. (2019). An Image Encryption Method Based on Chaos System and AES Algorithm. J. Supercomput..

[57] Li X., Yu C., Guo J. (2022). Multi-Image Encryption Method via Computational Integral Imaging Algorithm. Entropy.

[58] Neamah A.A., Shukur A.A. (2023). A Novel Conservative Chaotic System Involved in Hyperbolic Functions and Its Application to Design an Efficient Colour Image Encryption Scheme. Symmetry.

[59] Meng F.Q., Wu G. (2024). A Color Image Encryption and Decryption Scheme Based on Extended DNA Coding and Fractional-Order 5D Hyper-Chaotic System. Expert Syst. Appl..

[60] Zhang F., Wang X. (2024). Color Image Encryption Based on LSS-Type Coupled Mapped Lattice. IEEE Access.

[61] Deng X., Chen Z., Long B., Liu T., Wu X., Zheng Z., Zou S., Cao C. (2025). An Image Encryption Algorithm Based on a Novel 4D Hyperchaotic System and Improved Knight’s Tour Scrambling Algorithm. Phys. Scr..

[62] Yap W.-S., Phan C.-W., Yau W.-C., Heng S.-H. (2015). Cryptanalysis of a New Image Alternate Encryption Algorithm Based on Chaotic Map. Nonlinear Dyn..

[63] Man X., Song Y. (2023). Encryption of Color Images with an Evolutionary Framework Controlled by Chaotic Systems. Entropy.

[64] Cao Y., Song Y. (2024). Color Image Encryption Based on an Evolutionary Codebook and Chaotic Systems. Entropy.

[65] Sharma P.L., Gupta S., Nayyar A., Harish M., Gupta K., Sharma A.K. (2024). ECC Based Novel Color Image Encryption Methodology Using Primitive Polynomial. Multimed. Tools Appl..

[66] Shakhmetova G., Barlybayev A., Saukhanova Z., Sharipbay A., Raykul S., Khassenov A. (2024). Enhancing Visual Data Security: A Novel FSM-Based Image Encryption and Decryption Methodology. Appl. Sci..

[67] Byun H., Kim J., Jeong Y., Seok B., Gong S., Lee C. (2024). A Security Analysis of Cryptocurrency Wallets against Password Brute-Force Attacks. Electronics.

[68] Lin Z., Khetan A., Fanti G., Oh S. (2017). PacGAN: The Power of Two Samples in Generative Adversarial Networks. IEEE J. Sel. Areas Inf. Theory.

[69] Srivastava A., Valkov L., Russell C., Gutmann M.U., Sutton C. (2017). VEEGAN: Reducing Mode Collapse in GANs Using Implicit Variational Learning. Adv. Neural Inf. Process. Syst..

[70] Wang M., Lv S., You D., Wu X. (2024). Security Analysis of DeepKeyGen Based Medical Image Cryptosystem. 2024 9th International Conference on Intelligent Computing and Signal Processing, ICSP 2024.

[71] Dai L., Hu L., Chen L., Wang C., Lin F. (2024). An Image Double Encryption Based on Improved GAN and Hyper Chaotic System. IEEE Access.

[72] Dworkin M.J., Barker E., Nechvatal J.R., Foti J., Bassham L.E., Roback E., Dray J.F., National Institute of Standards and Technology (NIST) (2001). Advanced Encryption Standard (AES).

[73] Patidar V., Kaur G. (2023). A Novel Conservative Chaos Driven Dynamic DNA Coding for Image Encryption. Front. Appl. Math. Stat..

[74] Deb A., Renuka Devi S. (2025). A Hybrid Approach to Enhance The Image Security. 2025 5th International Conference on Intelligent Technologies, CONIT.

[75] Ahmed A., Abdelfattah M.G., Khalil A.T., Takieldeen A.E. (2024). Concealed Chosen Plaintext Attack on Multiple S-Boxes Based Image Encryption. J. Cybersecur. Inf. Manag..

[76] Liu H., Liu Y. (2014). Cryptanalyzing an Image Encryption Scheme Based on Hybrid Chaotic System and Cyclic Elliptic Curve. Opt. Laser Technol..

[77] Hu X., Zhang B., Al-Dossari M., El-Gawaad N.S.A., Rakhimzhanova M., Khan A.S. (2026). Robust Watermarking for Diffusion Models Using Error-Correcting Codes and Post-Quantum Key Encapsulation. Front. Phys..

